# Potential association between IL-12/23, IL-23, and IL-17 inhibitors and skin cancer: a pharmacovigilance study based on the FAERS database

**DOI:** 10.3389/fphar.2026.1944368

**Published:** 2026-10-22

**Authors:** Xing Jiang, Yunbei Huang, Min Xie, Li Peng, Mengyao Xu, Zhongyu Qi, Guojun Wang, Xiaofeng Pu

**Affiliations:** 1 Department of Pharmacy, People’s Hospital of Mianzhu, Mianzhu, Sichuan, China; 2 Department of Pharmacy, The Affiliated Hospital, Southwest Medical University, Luzhou, Sichuan, China; 3 School of Pharmacy, Southwest Medical University, Luzhou, Sichuan, China

**Keywords:** disproportionality analysis, FAERS, interleukin inhibitors, pharmacovigilance, skin cancer

## Abstract

**Background:**

Interleukin (IL)-12/23, IL-23, and IL-17 inhibitors are increasingly used for immune-mediated inflammatory diseases, yet their long-term risk of skin cancer remains unclear due to limited evidence from randomized controlled trials.

**Methods:**

We conducted a disproportionality analysis using individual case safety reports from the FDA Adverse Event Reporting System (FAERS) from 2004 to 2025 Q2. Reporting odds ratios (ROR) and information components (IC) were calculated to assess the association between IL-12/23, IL-23, and IL-17 inhibitors and skin cancer. Subgroup analyses by age and sex, time-to-onset analysis, Weibull modeling, and co-medication interaction analysis were further performed.

**Results:**

A total of 3,661 skin cancer reports were identified. Significant disproportionality signals were observed for most interleukin inhibitors, with the strongest associations for risankizumab (ROR 8.01, 95% CI 7.62–8.42; IC_025_, 2.88), tildrakizumab (ROR 5.81, 95% CI 4.13–8.19; IC_025_, 1.84), and ustekinumab (ROR 5.53, 95% CI 5.23–5.85, IC_025_, 2.35). IL-17 inhibitors showed weaker signals and were no longer significant in psoriasis-restricted sensitivity analyses. The association was more pronounced in elderly patients, especially for those aged >75 years. Weibull analysis indicated a wear-out failure pattern for ustekinumab and risankizumab, suggesting an increasing reporting frequency over time. Co-medication analyses did not reveal significant synergistic reporting signals.

**Conclusion:**

This pharmacovigilance study identified disproportionate reporting of skin cancer in reports involving IL-12/23 and IL-23 inhibitors, particularly risankizumab and ustekinumab, with weaker signals for IL-17 inhibitors. These findings represent signal detection from a spontaneous reporting database and highlight the need for dermatological monitoring in high-risk patients, warranting further prospective investigation to clarify clinical significance.

## Introduction

Skin cancer is one of the most common malignancies worldwide, with its incidence continuously rising in recent years (Wang et al., 2025). In addition, nonmelanoma skin cancer (excluding basal cell carcinoma) has become the most common cancer in Australia and New Zealand for both men and women, and in the United States for men (Bray et al., 2024). The main types of skin cancer include basal cell carcinoma (BCC), squamous cell carcinoma (SCC), and malignant melanoma (MM) (Linares et al., 2015). The development of skin cancer is influenced by a variety of factors, including ultraviolet (UV) radiation exposure, genetic susceptibility, and the use of immunosuppressive or immunomodulatory therapies. (Stern and PUVA Follow-Up Study, 2012; Garritsen et al., 2017; Newton-Bishop et al., 2020; Yu et al., 2023; Potestio et al., 2024). These therapies, which are often used in conditions like rheumatoid arthritis (RA), psoriasis, inflammatory bowel disease (IBD), and can alter immune responses and increase the risk of skin cancer (Scott et al., 2016; Asgari et al., 2017; Geller et al., 2018; Leonardi et al., 2019). In a retrospective study by Scott et al. (2016), the use of methotrexate (MTX) in patients with rheumatoid arthritis (RA) and inflammatory bowel disease (IBD) who had a history of non-melanoma skin cancer (NMSC) was associated with an increased risk of developing a second NMSC in RA patients. Additionally, several studies examining the role of TNF-inhibitors (TNFi) in skin cancer risk have indicated that the incidence of NMSC, particularly SCC, increased, with a notable association with prior phototherapy (Asgari et al., 2017; Geller et al., 2018). Furthermore, a recent extended safety analysis of adalimumab in patients with psoriasis found elevated standardized incidence rates for both SCC and melanoma (Leonardi et al., 2019).

However, newer biologic immunomodulatory agents, such as inhibitors of interleukins (IL)-12/23, IL-23, and IL-17, offer targeted treatments, but their potential impact on skin cancer risk, especially in vulnerable populations, remains unclear. These monoclonal antibodies have emerged as potent therapeutic tools, demonstrating superior efficacy in patients suffering from psoriasis, and related autoimmune pathologies (Kharouf and Gladman, 2024). The incidence of skin neoplasms in patients with psoriasis treated with ixekizumab (an IL-17 inhibitor) for ≤ 5 years was low, as demonstrated by an integrated safety analysis conducted by Smith et al. (2023). On skin cancers among psoriasis patients who were receiving ixekizumab in clinical trials. However, evidence from randomized controlled trials (RCTs) has been insufficient to definitively rule out long-term carcinogenic risks. RCTs are typically limited by short durations, relatively small sample sizes, and strict exclusion criteria that filter out patients with a history of malignancy, making them underpowered to detect rare or latency-dependent adverse events like skin cancer. While some meta-analyses suggest favorable safety profiles for IL-17 and IL-23 inhibitors regarding NMSC and melanoma (Krzysztofik et al., 2023; Wu et al., 2023), and a recent network meta-analysis found that these agents showed cancer risks broadly comparable with TNF inhibitors (Gibson et al., 2026), real-world data remains essential to validate these findings in a broader, unselected patient population over extended periods.

To address these limitations, this study uses the U.S. Food and Drug Administration Adverse Event Reporting System (FAERS), a large-scale pharmacovigilance database designed to support post-marketing safety surveillance (Weiss-Smith et al., 2011). Utilizing data from the FAERS database, this study aims to perform a disproportionality analysis to evaluate the potential association between IL-12/23, IL-23, and IL-17 inhibitors and the risk of skin cancer, while further exploring the underlying biological mechanisms.

## Methods

This study was reported according to The Reporting of A Disproportionality Analysis for Drug Safety Signal Detection Using Individual Case Safety Reports in PharmacoVigilance (READUS-PV) guideline (Fusaroli et al., 2024), and the detailed READUS-PV checklist was presented in Supplementary Table S12.

### Data source

Individual case safety reports (ICSRs) were obtained from the FDA Adverse Event Reporting System (FAERS) by downloading the raw quarterly ASCII data packages from the official U.S. FDA website, spanning from the database’s inception in 2004 through the second quarter of 2025 (2025 Q2). The raw datasets, which included all seven relational tables consisting of demographic (DEMO), drug (DRUG), adverse reaction (REAC), patient outcome (OUTC), therapy dates (THER), indications (INDI), and report sources (RPSR) files, were imported into R (version 4.4.0) for integrated processing and statistical analysis. To mitigate potential biases from redundant reporting, a multi-step deduplication algorithm was executed on the demographic (DEMO) datasets following FDA recommendations. Raw entries were sequentially sorted by case ID, FDA_DT, and PRIMARYID. For duplicate reports sharing the same case ID, only the most recent notification with the maximum FDA_DT was retained. If both case ID and FDA_DT were identical, the record with the highest PRIMARYID was preserved as the definitive case, and all other redundant variants were excluded. Drug standardization was performed by mapping brand names and synonymous terms to the generic names for IL-12/23 (ustekinumab), IL-23 (guselkumab, risankizumab, tildrakizumab), and IL-17 inhibitors (secukinumab, ixekizumab, brodalumab, bimekizumab). Adverse events were categorized using the Medical Dictionary for Regulatory Activities (MedDRA version 28.1) terminology, specifically utilizing the Broad scope of Standardised MedDRA Queries (SMQs) and Preferred Terms (PTs) related to skin malignant tumors. Data validation procedures for the time-to-onset analysis excluded reports with missing or illogical temporal information, such as cases where the event date (event_dt) preceded the therapy start date (start_dt) or contained invalid future dates. All data manipulation, including the merging of relational tables and the cleaning of missing values, was performed using R packages such as tidyverse and data.table to ensure a robust analytical cohort, with the detailed data processing flow illustrated in Figure 1.

**FIGURE 1:**
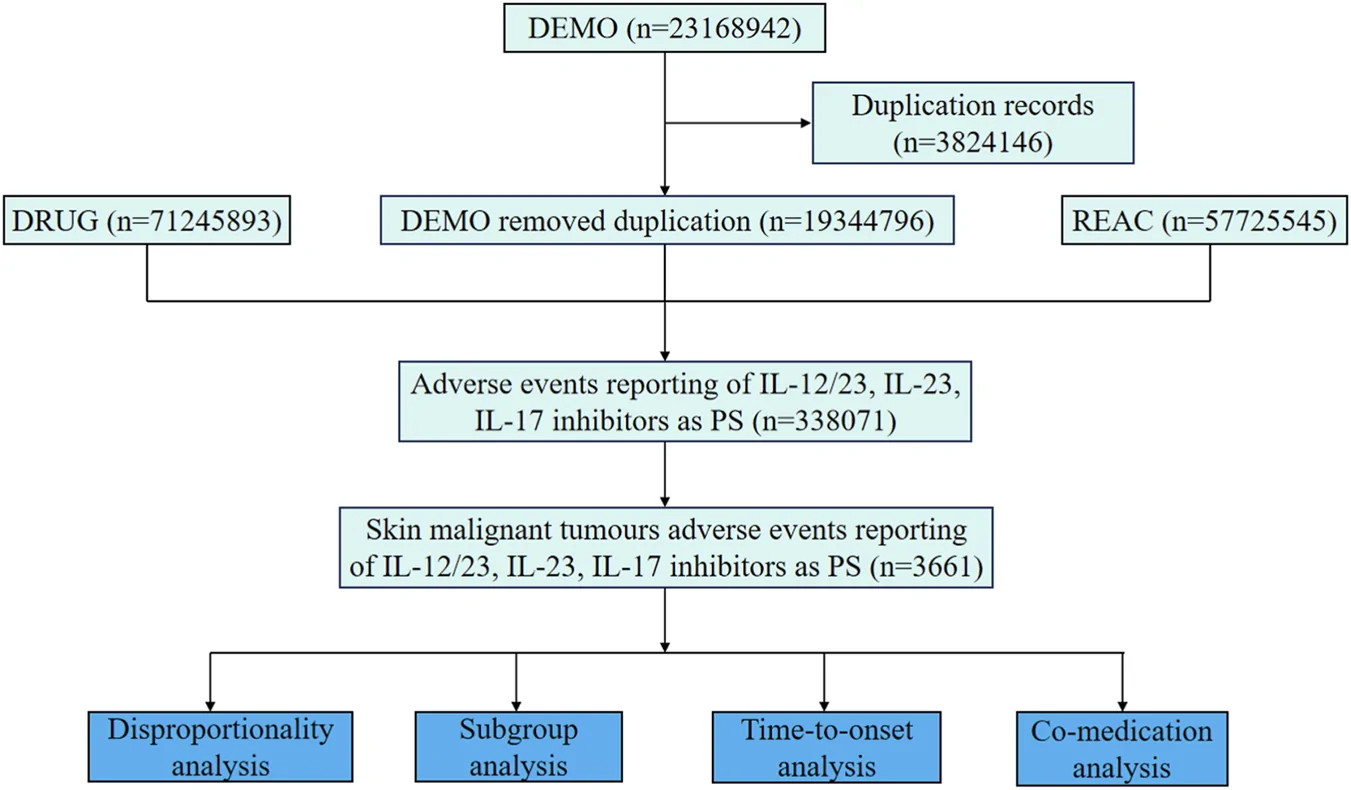
Data processing flow diagram. Flowchart of the FAERS data retrieval, deduplication, and case selection process for skin cancer adverse event reports associated with IL-12/23, IL-23, and IL-17 inhibitors.

### Adverse events and drug identification

In this study, all PTs included in the Broad scope of SMQ for “skin malignant tumors” were systematically retrieved and treated as adverse drug reactions of interest. These PTs included, but were not limited to, malignant melanoma, squamous cell carcinoma, squamous cell carcinoma of skin, basal cell carcinoma, and skin cancer. The complete list of PTs used for adverse event identification is provided in the Supplementary Table S1. Drug identification was performed using a combined search strategy of Medical Subject Headings (MeSH) terms and free-text terms, including generic names, brand names, and common spelling variants, to ensure comprehensive capture of all relevant FAERS reports. Reports were included when the drugs of interest were recorded as the primary suspect (PS) medication, thereby minimizing potential confounding bias from concomitant therapies and optimizing the specificity of the detected safety signals in alignment with standard pharmacovigilance methodologies.

### Statistical analysis

Disproportionality analyses were conducted to evaluate the association between biologic agents targeting the IL-12/23, IL-23, and IL-17 pathways and the reporting of skin malignant tumors in the FAERS database. Reporting odds ratio (ROR) and information component (IC) were calculated as measures of disproportionality. For each drug–adverse event pair, a two-by-two contingency table was constructed by comparing reports of skin malignant tumors associated with the drug of interest with all other adverse events reported for the same drug, as well as reports of skin malignant tumors associated with all other drugs in the database. The ROR was calculated together with its 95% confidence interval (CI). The IC, a Bayesian disproportionality metric derived from the Bayesian Confidence Propagation Neural Network (BCPNN), was also estimated. A signal was considered present when the number of cases was at least 3, the lower bound of the 95% CI of the ROR exceeded 1.0, and the IC_025_ (a lower limit of the 95% credibility interval of IC) exceeded zero. To minimize the risk of Type I errors inherent in multiple comparisons, we applied the Bonferroni correction to the calculated P-values. Specifically, the adjusted P-value was obtained by multiplying the raw P-value by the number of comparisons; an adjusted P < 0.05 was considered statistically significant. To ensure the robustness of our primary findings against terminology and indication biases, two sensitivity analyses were performed. In the first analysis we refined the target events by excluding non-specific, procedural, or ambiguous PTs from the broad SMQ “Skin Malignant Neoplasms”. In the second analysis, we combined this refined PT list with a restricted baseline population consisting strictly of a disease-matched cohort of patients with psoriasis or psoriatic arthritis. A third sensitivity analysis was conducted to address potential reporting bias by restricting the analysis to healthcare professional reports only (physicians, pharmacists, nurses, and other health professionals), excluding consumer/patient self-reports. To contextualize our findings, a comparative analysis was performed with other psoriasis treatments (TNF inhibitors, JAK inhibitors, and apremilast) in patients with psoriasis and psoriatic arthritis. Subgroup analyses were performed by stratifying reports according to age and sex. Age was categorized into predefined groups (i.e., <18, 18–65, 66–75 and >75 years), and sex was classified as male or female, based on information available in the DEMO file. Disproportionality measures were recalculated within each subgroup to explore potential effect modification. Reports with missing age or sex information were excluded from the corresponding subgroup analyses. For agents with sufficient reports (ustekinumab, secukinumab, and risankizumab), a calendar period-stratified analysis was performed. Each agent was stratified into early post-marketing period (first 5 years after approval) and late post-marketing period (≥6 years after approval) to evaluate whether signals persisted beyond the early post-approval period.

Time-to-onset (TTO) analysis was conducted to assess the temporal association between initiation of biologic therapy and the occurrence of skin malignant tumors. TTO was defined as the interval (in days) between the therapy start date and the adverse event onset date, using information from the THER and DEMO files of the FAERS database. Reports with missing, incomplete, or implausible dates, including negative time intervals, were excluded. TTO distributions for each biologic agent were summarized using the median and interquartile range (IQR), and cumulative distribution curves were constructed. Differences in TTO among drugs were compared using the Kruskal–Wallis test. Besides, Weibull shape parameter (WSP) analysis was used to evaluate the reporting trend of AEs over time. To ensure statistical robustness, the analysis was restricted to drug–AE pairs with at least 20 cases with valid time-to-onset data.

Co-medication analysis was performed to explore the potential impact of concomitant medications on the reporting of skin malignant tumors. Three biologic agents (risankizumab, secukinumab, and ustekinumab) were included in this analysis, as they had sufficient numbers of reports for stable estimation. In addition, seven concomitant medications previously reported (Jiyad et al., 2016; Arana et al., 2021; Semaka and Salopek, 2022; Hashizume et al., 2023; Potestio et al., 2024) in the literature to be potentially associated with an increased risk of skin cancer (azathioprine, cyclosporine, hydrochlorothiazide, infliximab, tacrolimus, methotrexate, and apremilast) were selected for evaluation. The interaction between each biologic agent and the selected co-medications was assessed using the Ω shrinkage measure, and the lower bound of the 95% credibility interval (Ω025) was calculated. A negative Ω025 value was interpreted as no evidence of a synergistic reporting signal. This analysis was exploratory and intended to aid interpretation of disproportionality findings rather than to infer causality.

All statistical tests were two-sided, and a p-value <0.05 was considered statistically significant. Confidence intervals and credibility intervals were calculated at the 95% level for all estimates.

## Results

### Descriptive analysis

We identified 3,661 adverse event (AE) reports associated with the included biologics targeting the IL-12/23, IL-23, and IL-17 pathways from the FAERS database (Table 1). Among these, risankizumab accounted for the largest number of reports (1,525 cases), followed by ustekinumab (1,182 cases) and secukinumab (639 cases), whereas bimekizumab (20 cases) and brodalumab (21 cases) were associated with relatively few reports. The number of male patients and female patients were comparable (47.9% vs. 45.8%), and the remaining reports lacked sex information. Most AEs were reported in adult patients who aged 18–65 years, while reports of patients aged <18 years or >85 years were uncommon. Reports were submitted by both healthcare professionals and consumers, with consumers accounting for 54.4% and healthcare professionals for 35.3% of all reports, and the United States was the primary reporting country (Table 1). To contextualize the cumulative reporting volumes across different biologics, the chronological FDA approval years for each agent were retrieved. Among the included interleukin inhibitors, ustekinumab was the earliest approved agent (2009), followed by secukinumab (2015), ixekizumab (2016), brodalumab (2017), guselkumab (2017), tildrakizumab (2018), risankizumab (2019), and bimekizumab (2023). As shown in Figure 2, the annual number of AE reports increased steadily following market approval of these agents. Among these AE reports, an observed rise was noted for risankizumab and secukinumab in recent years, and ustekinumab showed a more gradual but sustained increase. Recently approved agents, such as bimekizumab, exhibited lower reporting volumes, which may be attributed to their limited time on the market.

**TABLE 1:** Study characteristics.

Categories	USE N (%)	GUS N (%)	RIS N (%)	TIL N (%)	SEC N (%)	IXE N (%)	BRO N (%)	BIM N (%)	Total N (%)
Reports	1182	107	1525	33	639	134	21	20	3661
FDA approval year	2009	2017	2019	2018	2015	2016	2017	2023	
Sex									
Female	508 (43.0)	33 (30.8)	737 (48.3)	14 (42.4)	302 (47.3)	62 (46.3)	9 (42.9)	12 (60.0)	1677 (45.8)
Male	562 (47.5)	46 (43.0)	756 (49.6)	19 (57.6)	292 (45.7)	62 (46.3)	12 (57.1)	6 (30.0)	1755 (47.9)
Missing	112 (9.5)	28 (26.2)	32 (2.1)	0 (0)	45 (7.0)	10 (7.5)	0 (0)	2 (10.0)	229 (6.3)
Age									
<18 years	34 (2.9)	1 (0.9)	0 (0)	0 (0)	1 (0.2)	0 (0)	0 (0)	1 (5.0)	37 (1.0)
18–64.9 years	412 (34.9)	27 (25.2)	311 (20.4)	10 (30.3)	165 (25.8)	64 (47.8)	12 (57.1)	3 (15.0)	1004 (27.4)
65–85 years	312 (26.4)	27 (25.2)	376 (24.7)	16 (48.5)	110 (17.2)	29 (21.6)	6 (28.6)	3 (15.0)	879 (24.0)
>85 years	8 (0.7)	1 (0.9)	21 (1.4)	0 (0)	1 (0.2)	2 (1.5)	1 (4.8)	0 (0)	34 (0.9)
Missing	416 (35.2)	51 (47.7)	817 (53.6)	7 (21.2)	362 (56.7)	39 (29.1)	2 (9.5)	13 (65.0)	1707 (46.6)
Outcomes									
Death	17 (1.4)	2 (1.9)	3 (0.2)	0 (0)	17 (2.7)	3 (2.2)	0 (0)	0 (0)	42 (1.1)
Disability	3 (0.3)	0 (0)	0 (0)	0 (0)	2 (0.3)	0 (0)	0 (0)	0 (0)	5 (0.1)
Hospitalization	103 (8.7)	6 (5.6)	64 (4.2)	3 (9.1)	67 (10.5)	6 (4.5)	2 (9.5)	2 (10.0)	253 (6.9)
Life-threatening	26 (2.2)	2 (1.9)	3 (0.2)	3 (9.1)	11 (1.7)	6 (4.5)	2 (9.5)	0 (0)	53 (1.4)
Missing	171 (14.5)	7 (6.5)	1 (0.1)	0 (0)	18 (2.8)	17 (12.7)	0 (0)	0 (0)	214 (5.8)
Other serious illness	861 (72.9)	90 (84.1)	1454 (95.3)	27 (81.8)	524 (82.0)	102 (76.1)	17 (81.0)	18 (90.0)	3094 (84.5)
Country of the reporter (TOP 3)									
​	US 406 (34.3)	US 46 (43.0)	US 1181 (77.4)	US 16 (48.5)	US 201 (31.5)	US 70 (52.2)	US 6 (28.6)	US 11 (55.0)	US 1937 (52.9)
​	GB 255 (21.6)	FR 10 (9.3)	CA 72 (4.7)	DE 5 (15.2)	CA 60 (9.4)	DE 7 (5.2)	CA 5 (23.8)	CA 4 (20.0)	GB 346 (9.5)
​	CA 163 (13.8)	DE 7 (6.5)	GB 30 (2.0)	GB 5 (15.2)	DE 51 (8.0)	CA 5 (3.7)	FR 2 (9.5)	DE 2 (10.0)	CA 316 (8.6)
Reporter									
Consumer	296 (25.0)	12 (11.2)	1376 (90.2)	10 (30.3)	214 (33.5)	69 (51.5)	5 (23.8)	10 (50.0)	1992 (54.4)
Health professional	193 (16.3)	20 (18.7)	58 (3.8)	13 (39.4)	112 (17.5)	19 (14.2)	5 (23.8)	4 (20.0)	424 (11.6)
Pharmacist	28 (2.4)	5 (4.7)	1 (0.1)	2 (6.1)	14 (2.2)	4 (3.0)	0 (0)	0 (0)	54 (1.5)
Physician	375 (31.7)	62 (57.9)	88 (5.8)	8 (24.2)	237 (37.1)	27 (20.1)	10 (47.6)	6 (30.0)	813 (22.2)
Missing	290 (24.5)	8 (7.5)	2 (0.1)	0 (0)	62 (9.7)	15 (11.2)	1 (4.8)	0 (0)	378 (10.3)

USE, ustekinumab; GUS, guselkumab; RIS, risankizumab; TIL, tildrakizumab; SEC, secukinumab; IXE, ixekizumab; BRO, brodalumab; BIM, bimekizumab; US, united states; GB, united kingdom; CA, canada; FR, france; DE, germany.

**FIGURE 2:**
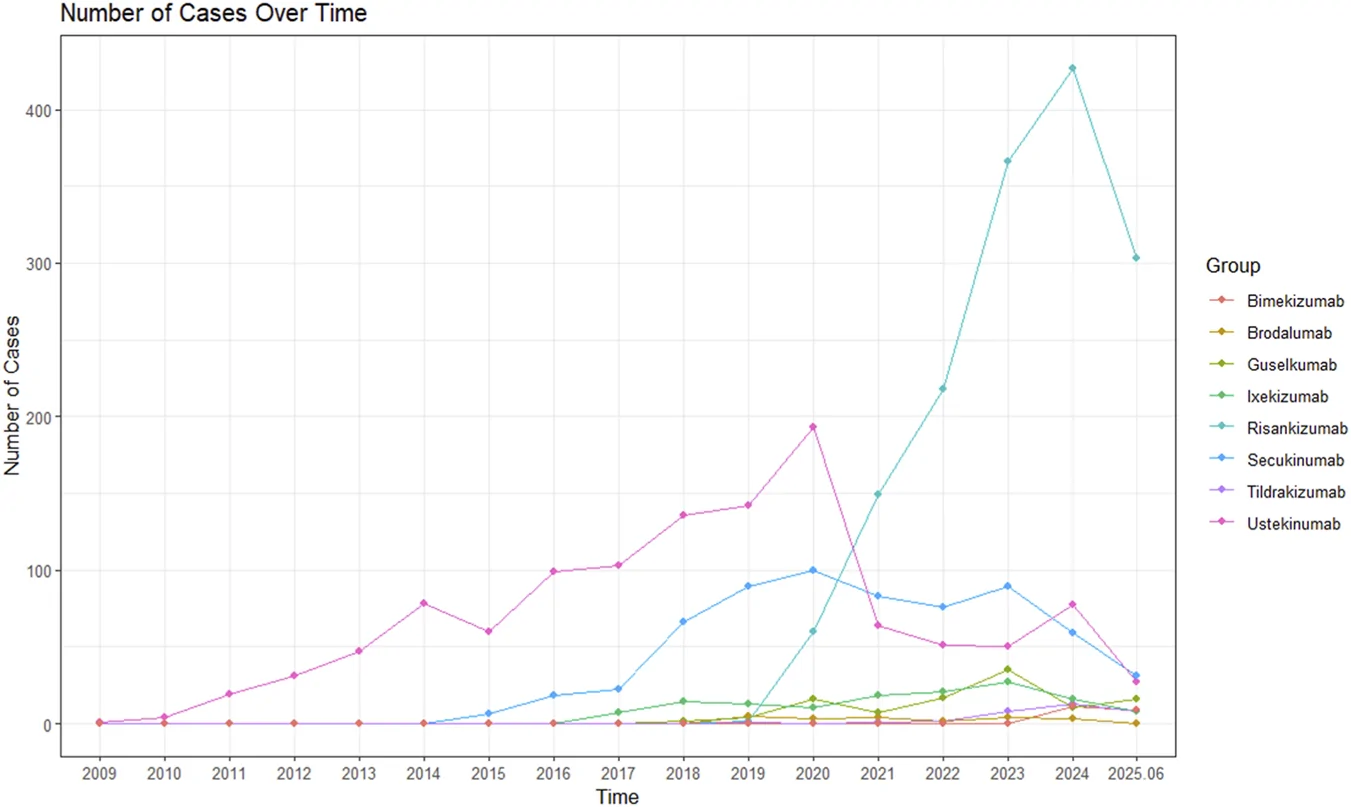
Annual distribution of skin cancer adverse event reports. Number of skin cancer reports associated with each interleukin inhibitor by year, showing the reporting trends following market approval of each agent.

### Disproportionality analysis

We conducted a disproportionality analysis using RORs and the lower bound of the information component (IC_025_) to identify potential safety signals, and the results were presented in Figure 3; Supplementary Table S2. Overall, a significant disproportionality signal was observed for the included interleukin inhibitors (ROR 3.31, 95% CI: 3.20 to 3.42; IC_025_, 1.62). Among IL-12/23 and IL-23 inhibitors, risankizumab was associated with the highest ROR (8.01, 95% CI: 7.62–8.42), and a corresponding IC_025_ of 2.88. Potential safety signals were also detected for ustekinumab (ROR 5.53, 95% CI: 5.23 to 5.85; IC025, 2.35), guselkumab (ROR 1.57, 95% CI: 1.31 to 1.89; IC_025_, 0.37) and tildrakizumab (ROR 5.81, 95% CI: 4.13 to 8.19; IC_025_, 1.84). For IL-17 inhibitors, secukinumab (ROR 1.18, 95% CI: 1.09 to 1.27; IC_025_, 0.12), ixekizumab (ROR 1.72, 95% CI: 1.45 to 2.03; IC_025_, 0.53), and brodalumab (ROR 2.31, 95% CI: 1.53 to 3.47; IC_025_, 0.54) showed statistically significant signals, whereas no signal was detected for bimekizumab (ROR 0.80, 95% CI: 0.51 to 1.24; IC_025_, −0.94).

**FIGURE 3:**
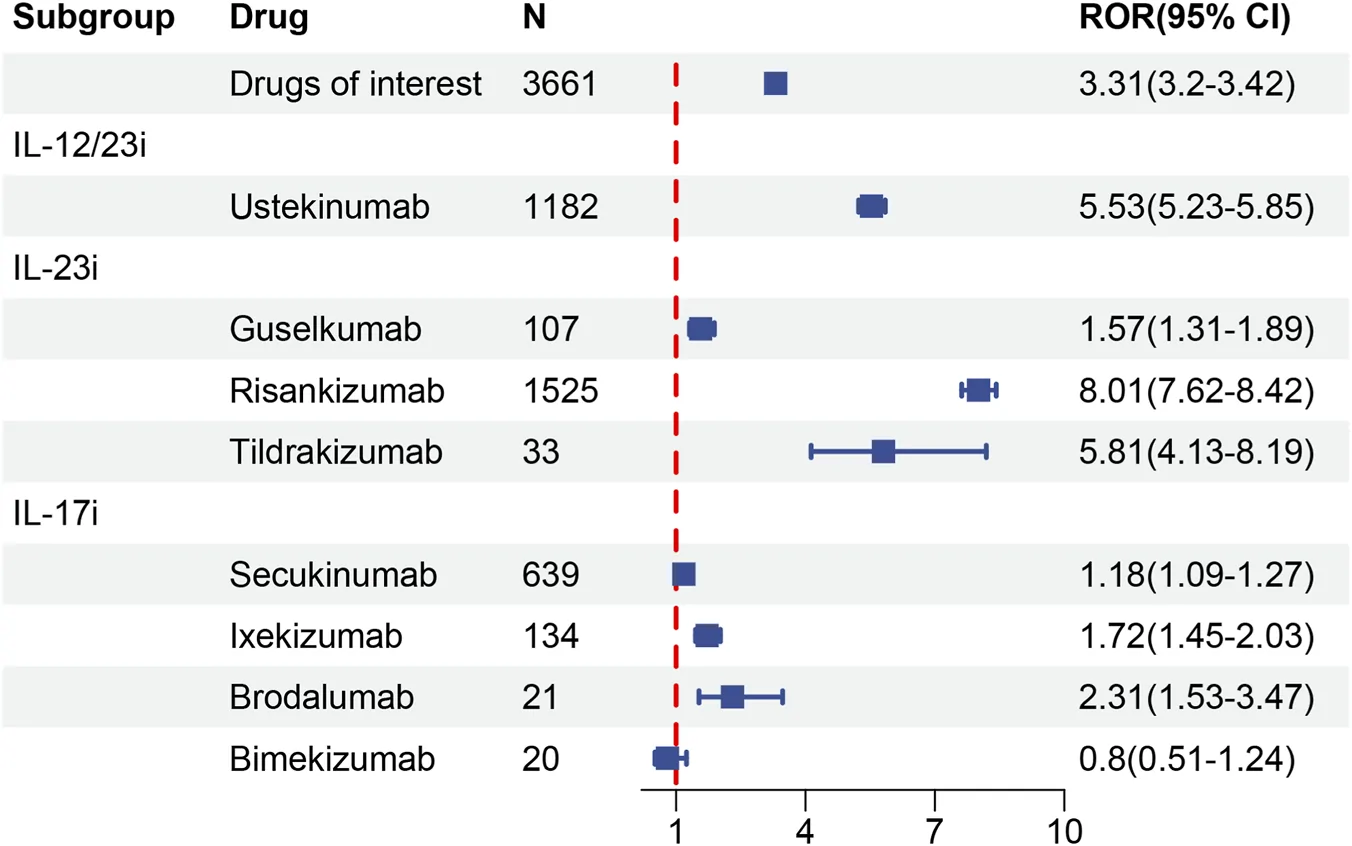
Forest plot of disproportionality analysis. RORs and 95% CI for the association between IL-12/23, IL-23, and IL-17 inhibitors and skin cancer, based on the FAERS database. ROR, Reporting odds ratios; CI, confidence intervals.

Sensitivity analyses confirmed that our primary results were generally robust. In the first analysis, after excluding 29 non-specific PTs (Supplementary Table S3), the overall signal for Interleukin inhibitors remained significant (ROR = 3.53; 95% CI: 3.41–3.65). Strong signals were also sustained for nearly all individual drugs, with risankizumab (ROR = 8.48) and ustekinumab (ROR = 5.87) leading the way; however, bimekizumab showed no signal. In the second analysis, where we restricted the background to the psoriasis cohort (Supplementary Table S4), the class-level signal stayed clearly positive (ROR = 2.00; 95% CI: 1.91–2.10), though with a moderate attenuation. At the individual-agent level, a clear split emerged. Ustekinumab (ROR = 4.37) and risankizumab (ROR = 4.41) continued to show strong independent safety signals, whereas all pure IL-17 inhibitors (secukinumab, ixekizumab, and brodalumab) lost their previously positive signals entirely. Bimekizumab remained negative throughout (ROR = 0.51). In sensitivity analyses restricted to healthcare professional reports, the overall ROR remained stable (ROR 3.26, 95% CI 3.11–3.42), and all significant signals persisted (Supplementary Table S5). Notably, the ROR for risankizumab decreased from 8.01 to 5.03, suggesting that consumer self-reports may have contributed to signal inflation for this agent; however, the signal remained statistically significant.

Subgroup analyses stratified by age and sex revealed distinct signal patterns across the investigated biologic classes, as shown in Supplementary Table S6. The IL-12/23 inhibitor (ustekinumab) demonstrated consistently strong disproportionality signals across all subgroups; notably, when evaluating the elderly population with finer granularity, the signal strength escalated with advancing age, reaching its peak in patients aged >75 years (ROR 11.40, 95% CI 9.45–13.75) compared to those aged 66–75 years (ROR 9.84, 95% CI 8.66–11.18). Within the IL-23 inhibitor class, risankizumab showed robust signals in both age groups (RORs >6.0) and both sexes, while guselkumab exhibited a significant signal only in the elderly population (66–75 years, ROR 2.35, 95% CI 1.48 to 3.74; >75 years, ROR 3.47, 95% CI 1.92–6.27). Regarding IL-17 inhibitors, ixekizumab maintained significant signals across most subgroups (RORs >1.6), whereas secukinumab showed a significant association primarily in elderly patients, displaying an increasing trend from the 66–75 age group (ROR 1.62, 95% CI 1.32–1.99) to the >75 age group (ROR 1.79, 95% CI 1.25–2.54). Signals for brodalumab and bimekizumab were more variable, likely limited by smaller case volumes. Overall, the association with skin cancer reports was more pronounced in elderly patients (>65 years) for nearly all biologics showing positive signals, with the highest signal intensities consistently observed in the oldest-old cohort (>75 years). We further characterized skin cancer related adverse event profiles, and conducted a preferred term (PT)–level analysis based on the top 10 PTs with the highest number of reports. The presence and magnitude of signals differed across drug classes, with IL-12/23 and IL-23 inhibitors showing signals across a broader range of PTs than IL-17 inhibitors. In addition, these signals were predominantly observed in basal cell carcinoma, malignant melanoma, and squamous cell carcinoma, as shown in Figure 4; Supplementary Table S7. To compare with the signals observed for IL-12/23, IL-23, and IL-17 inhibitors, we analyzed other psoriasis treatments, including TNF inhibitors, JAK inhibitors, and apremilast, in patients with psoriasis and psoriatic arthritis (Supplementary Table S8). The pooled estimates for JAK inhibitors (ROR 1.08, 95% CI 0.88–1.31; IC025–0.18) and TNF inhibitors (ROR 0.95, 95% CI 0.90–0.99; IC_025_–0.11) did not show significant signals. Apremilast also showed no signal (ROR 0.38, 95% CI 0.33–0.42; IC_025_–1.48). However, individual drugs showed heterogeneous results, with significant signals observed for infliximab (ROR 3.97), golimumab (ROR 2.44), and upadacitinib (ROR 1.84). In addition, calendar period-stratified analysis was performed for agents with sufficient reports (Supplementary Table S9). Ustekinumab showed a two-fold stronger signal in the early post-marketing period (ROR 12.58) compared with the late period (ROR 5.04). Secukinumab showed weak signals throughout, with the late period signal becoming non-significant. Risankizumab showed a stronger signal in the late period (ROR 8.68) compared with the early period (ROR 6.44).

**FIGURE 4:**
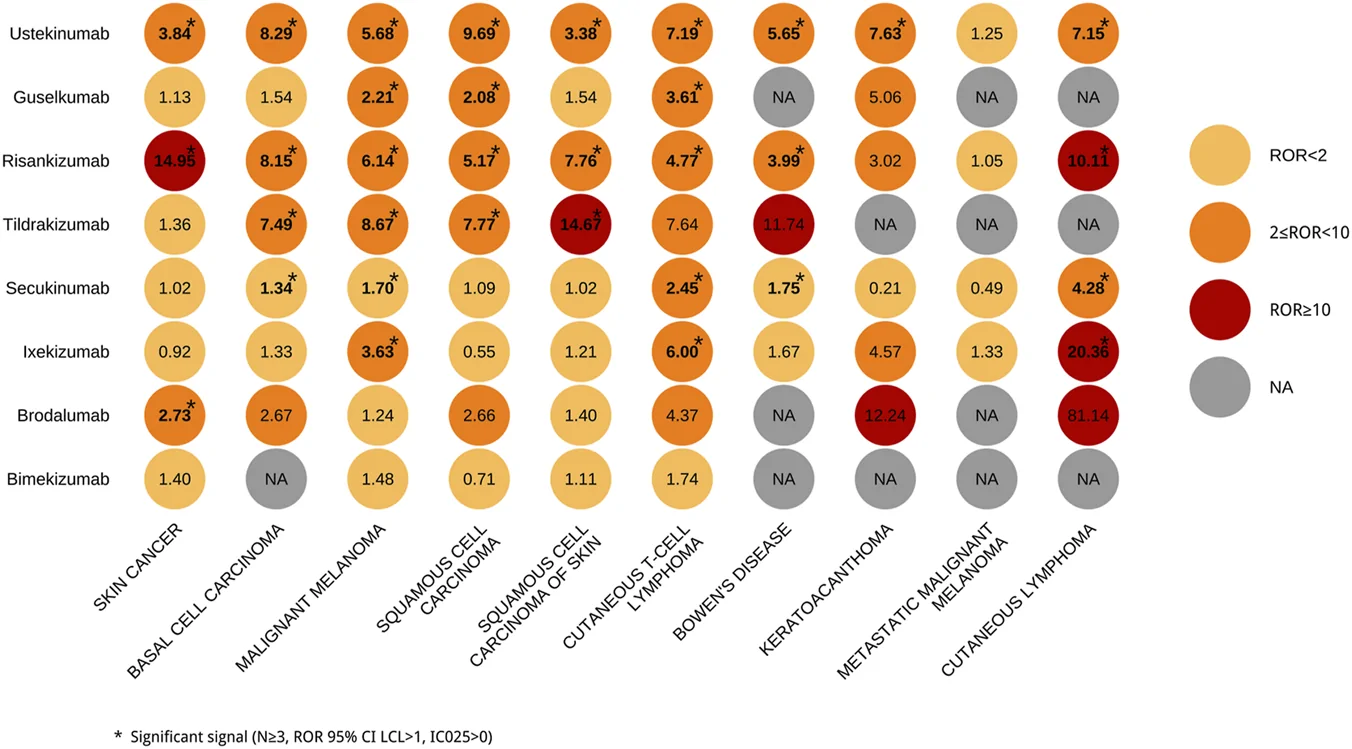
Heatmap of preferred term-level disproportionality signals. Distribution and magnitude of disproportionality signals across the top 10 skin cancer-related Preferred Terms for each interleukin inhibitor. Color intensity represents ROR values. Bold values with asterisks (*) indicate significant signals (N ≥ 3, ROR 95% CI lower bound >1, and IC_025_ > 0); exact RORs with 95% CIs are provided in Supplementary Table S7. ROR, reporting odds ratios; NA, denotes not applicable due to zero cases; CI, confidence interval; IC_025_, the lower bound of the 95% credibility interval for the Information Component.

### Time-to-onset analyses

As shown in Figure 5, cumulative time-to-onset curves varied across the involved biologic agents. The shortest median time to skin cancer onset was observed for ixekizumab (216 days; interquartile range [IQR], 135–485.5), followed by secukinumab (259.5 days; IQR, 95.8–577), risankizumab (258 days; IQR, 105–497.2), brodalumab (295 days; IQR, 234–585.5) and bimekizumab (306 days; IQR, 184–412.5). In contrast, longer median times to onset were observed for tildrakizumab (338.5 days; IQR, 205.2–516.8), ustekinumab (370.5 days; IQR, 151.5–604.8), and guselkumab (375 days; IQR, 251–654). The Kruskal–Wallis test indicated a statistically significant difference in time-to-onset distributions among the included interleukin inhibitors (P = 0.022).

**FIGURE 5:**
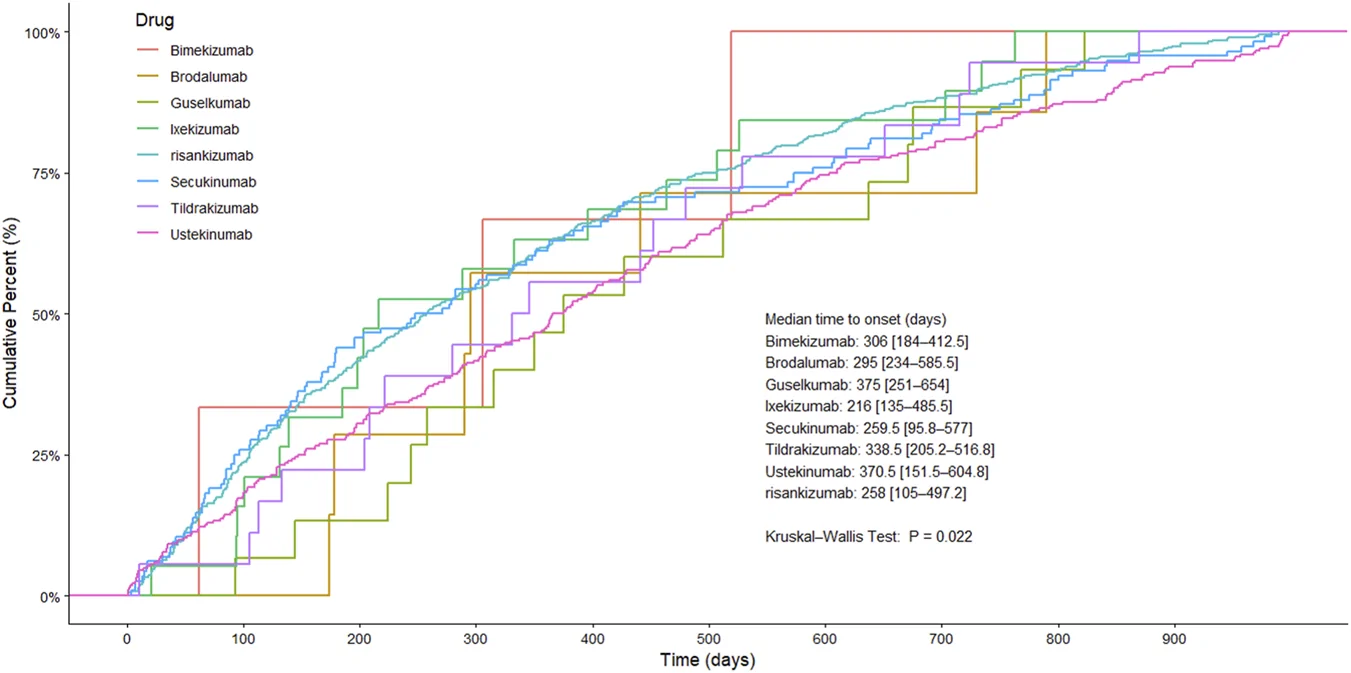
Cumulative time-to-onset curves. Cumulative incidence curves of time from biologic therapy initiation to skin cancer onset for each interleukin inhibitor. The Kruskal–Wallis test indicated a significant difference among groups (P = 0.022).

We further performed a weibull modeling for agents with sufficient case numbers (Supplementary Table S10). The results showed that secukinumab (n = 116) had a shape parameter close to 1 (β = 1.10; 95% CI, 0.98–1.30), indicating a random failure pattern with an approximately constant reporting rate over time (α = 352.73 days; 95% CI, 295.39–412.50). However, ustekinumab (n = 272) and risankizumab (n = 392) demonstrated wear-out failure patterns with shape parameters and associated 95% CI greater than 1 (ustekinumab: β = 1.22 [95% CI, 1.12–1.35], α = 429.76 days; risankizumab: β = 1.18 [95% CI, 1.10–1.28], α = 344.36 days), suggesting a statistical trend of an increasing reporting pattern over time within this dataset. Weibull parameters could not be estimated for the remaining agents because they did not meet the sample size threshold (n < 20).

### Co-medication analysis

We also conducted a co-medication analysis to explore the potential impact of concomitant medications on skin cancer reporting signals for risankizumab, secukinumab, and ustekinumab. Seven medications previously reported in the literature to be potentially associated with an increased risk of skin cancer (azathioprine, cyclosporine, hydrochlorothiazide, infliximab, tacrolimus, methotrexate, and apremilast) were evaluated. Disproportionality signals for skin cancer were identified in reports involving most of these drugs. However, the Ω shrinkage analysis revealed no evidence of synergistic reporting signals between the three biologics and the selected concomitant medications, as all Ω_025_ values were negative (see Supplementary Table S11 for the ROR, IC_025_, and Ω_025_ values for each medication).

## Discussion

The main findings of this study showed that most IL-12/23, IL-23, and IL-17 inhibitors were associated with disproportionally higher reporting of skin cancer, with the exception of bimekizumab. Notably, IL-12/23 and IL-23 inhibitors, particularly risankizumab and ustekinumab, exhibited substantially stronger signals than IL-17 inhibitors, and these independent safety signals remained robust even after refining terminology and restricting the baseline population to the psoriasis cohort in sensitivity analyses, whereas the positive signals for pure IL-17 inhibitors faded upon cohort restriction. Subgroup analyses further revealed that the association was more pronounced in elderly patients, particularly those aged over 75 years, and varied across drug classes and patient characteristics. Time-to-onset and Weibull analyses suggested heterogeneous temporal reporting profiles. Ustekinumab and risankizumab showed a wear-out failure pattern characterized by a statistically increasing reporting pattern over time, while secukinumab followed a stable reporting pattern. Co-medication analyses did not identify significant synergistic reporting signals, indicating that the observed signals were unlikely to be driven by concomitant therapies. When compared with other psoriasis treatments, the results were heterogeneous. We found that while the pooled estimates for JAK inhibitors and TNF inhibitors did not show significant skin cancer signals, individual drugs such as infliximab, golimumab, and upadacitinib did exhibit significant signals. This heterogeneity within drug classes highlights the complexity of interpreting disproportionality data. Nevertheless, the apparently stronger signals for IL-12/23 and IL-23 inhibitors should be interpreted with caution. In the psoriasis-restricted analyses, ustekinumab (ROR 4.37) and risankizumab (ROR 4.41) were similar in magnitude to infliximab (ROR 3.97), while guselkumab showed no signal (ROR 0.78). These findings suggest that the stronger signals observed in the overall population may not represent genuine differences in risk profiles, and methodological explanations, including confounding by indication and differences in reporting patterns, should be considered. Calendar period-stratified analysis (Supplementary Table S9) may also inform signal interpretation. The ustekinumab signal was markedly stronger in the early post-marketing period (ROR 12.58 vs. 5.04) than in the late period, a pattern compatible with stimulated reporting shortly after approval (Weiss-Smith et al., 2011), whereas risankizumab showed the reverse pattern despite only 2 years of late-period data. Importantly, signals persisted beyond the early post-approval period for most agents, indicating that early stimulated reporting alone does not fully account for the observed associations.

Previously, two meta-analyses evaluated the risk of melanoma and NMSC in patients who received biologic therapy in comparison to those not receiving such therapy. The findings suggested that the risk of skin cancer increased to a clinically significant extent; however, this association was primarily observed in patients treated with TNF-α inhibitors (Esse et al., 2020; Liu et al., 2021). A recent meta-analysis showed that the incidence rates (IRs) of melanoma were consistent across patients treated with newer medications (IL-17 inhibitors, IL-23 inhibitors, and JAK inhibitors) and were similar to previously published findings in patients receiving TNF-α inhibitors (Krzysztofik et al., 2023). In addition, a research showed that TNF-α inhibitors and JAK inhibitors show a slightly increased risk of skin cancer, other treatments, such as anti-integrin agents and interleukin inhibitors, appear to have lower risks (Bencardino et al., 2025). Although current evidence indicates that interleukin inhibitors (particularly IL-17, IL-12/23 and IL-23 inhibitors) do not significantly increase the risk of skin cancer, a review noted that NMSC was excluded from several analyses, and long-term data are still lacking (Kreher et al., 2023a). Therefore, pharmacovigilance studies are of great importance, especially for the detection of rare serious adverse reactions to newer drugs.

In this study, we found that most of the included interleukin inhibitors demonstrated significant disproportionality signals of skin cancer. Notably stronger signals were observed for IL-12/23 and IL-23 inhibitors, compared with IL-17 inhibitors. IL-12 and IL-23 share a common p40 subunit, enabling p40-targeted agents to concurrently neutralize both cytokines (Teng et al., 2015). Despite this structural homology, they govern distinct immunological cascades. IL-12 drives Th1 cell differentiation to elicit IFN-γ-mediated anti-tumor immunity, whereas IL-23 sustains Th17 cell expansion and induces IL-17A/F secretion. This functional divergence positions IL-23 as the critical upstream regulator of the pro-inflammatory IL-23/IL-17 signaling axis (Teng et al., 2015). While these IL12/23 dual inhibitors block the IL-23, which has been associated with tumor promotion in preclinical studies, they simultaneously block IL-12, which has been associated with anti-tumor immunity. IL-12 plays a key role in preventing tumor initiation and growth by activating NK cells and CD8^+^ T cells to produce IFN-γ (Dunn et al., 2006). In addition, mice lacking the IL-12-specific p35 subunit are notably more susceptible to carcinogen-induced skin papilloma formation (Langowski et al., 2006). IL-23 has been associated with tumor promotion in preclinical studies, including enhanced tumor incidence, growth, and angiogenesis (Zhao et al., 2021).

In preclinical studies, mice deficient in the IL-23p19 subunit are strongly protected against carcinogen-induced skin papilloma formation and fibrosarcomas (Langowski et al., 2006; Teng et al., 2010; Zhao et al., 2021). Thus, specific neutralization of IL-23 may eliminate its pro-tumor effects without compromising IL-12, potentially favoring skin cancer prevention. IL-17 signaling strongly stimulates this epidermal proliferation. Animal models have proven that defects in IL-17 signaling (such as an IL-17RC knockout or IL-17RA mutation) significantly inhibit epidermal hyperplasia and completely abolish the development of carcinogen-induced skin papillomas. Therefore, inhibiting IL-17 may be beneficial for reducing the risk of such skin tumors (Jin et al., 2022). These mechanistic insights, together with the observation that IL-12/23i and IL-23i signals remained consistent whereas IL-17i signals were no longer significant in psoriasis-restricted analyses, may help explain the observed signal gradient across drug classes. The strongest signals were detected for p40-targeted IL-12/23 inhibitors, which block both IL-12 and IL-23. Intermediate signals were observed for p19-targeted IL-23 inhibitors, which selectively inhibit IL-23. IL-17 inhibitors, which act further downstream in the inflammatory cascade, showed the weakest or non-significant signals. This gradient is consistent with the mechanistic differences described above. IL-23 drives chronic inflammation and tumor promotion, whereas IL-17 primarily mediates acute inflammatory responses. The null finding for bimekizumab, however, warrants caution given its limited market exposure. Notably, heterogeneity within the IL-23 inhibitor class was observed, with risankizumab showing substantially stronger signals than guselkumab and tildrakizumab. This heterogeneity is more likely explained by methodological factors such as differences in market entry time, reporting patterns, and patient populations, rather than by biological differences between agents. These findings should therefore be interpreted with caution, and class-level estimates may be preferable for conclusions about IL-23 inhibitors as a class. Together, these observations suggest that mechanistic plausibility differs across drug classes. The strong and consistent signals for IL-12/23 inhibitors align with the loss of IL-12-mediated tumor immunosurveillance described above and are therefore biologically plausible. For selective IL-23 inhibitors, however, the situation appears paradoxical, because the preclinical evidence reviewed above implies that IL-23 blockade should be protective rather than harmful. Several factors may reconcile this discrepancy. IL-23 exerts context-dependent, dual pro- and anti-tumor functions, so the net consequence of chronic blockade on cutaneous immune surveillance remains poorly defined (Ahmed and Sonnenberg, 2026). Chronic blockade may also unmask pre-existing subclinical lesions, consistent with the short time-to-onset intervals observed here (Linares et al., 2015). In addition, residual confounding and reporting biases may contribute to the observed signals. These signals should therefore be regarded as hypothesis-generating, and the apparent conflict between preclinical expectations and real-world reporting itself warrants mechanistic investigation.

The subgroup and stratified analyses provide further insights into the heterogeneity of skin cancer reporting associated with biologic therapies. Stronger signals observed in elderly patients (especially >75) may reflect age-related immunosenescence and cumulative environmental carcinogenic exposure, providing biological context for the observed reporting pattern (Liu et al., 2023; Yu et al., 2023). The sensitivity analysis restricted to healthcare professional reports demonstrated that the main findings were robust, although the signal for risankizumab was attenuated. This suggests that consumer self-reports, which accounted for 90.2% of risankizumab reports, may have inflated the disproportionality signal for this agent. However, the signal remained significant even after excluding consumer reports, suggesting that the signal persists independently of consumer reporting bias. At the preferred term level, signals were mainly concentrated in basal cell carcinoma, squamous cell carcinoma, and malignant melanoma, consistent with the most common skin malignancies observed in immunosuppressed populations (Kreher et al., 2023b), while the broader distribution of signals across multiple preferred terms for IL-12/23 and IL-23 inhibitors may indicate a wider influence on cutaneous oncogenic processes (Zhao et al., 2021). Additionally, differences in time-to-onset and temporal reporting patterns suggest variability in the underlying mechanisms of tumor development. The wear-out pattern was observed for ustekinumab and risankizumab, reflecting a statistical trend of an increasing reporting pattern over time within this dataset, rather than biological cumulative toxicity**.** Whereas the approximately constant reporting frequency identified for secukinumab suggests a distinct temporal dynamic (Sauzet and Cornelius, 2022), possibly reflecting differences in downstream pathway inhibition and immune modulation intensity. The median time-to-onset was 216–375 days. Given that solid tumors generally take years to arise via *de novo* carcinogenesis, this relatively short interval suggests these events may not reflect true new malignancies. Rather, biologic-induced immunosuppression may unmask or speed the progression of pre-existing subclinical skin lesions, pointing to impaired immune surveillance more than to primary oncogenic initiation (Linares et al., 2015). Based on this, baseline dermatological evaluation prior to biologic initiation and early surveillance within the first 6–12 months of treatment may be warranted, particularly for IL-12/23 and IL-23 inhibitors which showed the strongest signals. Importantly, co-medication analyses did not reveal significant synergistic reporting signals after shrinkage adjustment, indicating that the observed associations are unlikely to be driven by concomitant therapies. Taken together, these findings highlight the complexity and heterogeneity of biologic agents associated skin cancer reporting and underscore the importance of considering patient characteristics, drug class, and treatment duration in signal interpretation.

Notably, current evidence has not consistently demonstrated an increased risk of skin cancer associated with interleukin inhibitors (Krzysztofik et al., 2023; Bencardino et al., 2025). In contrast, our pharmacovigilance analysis based on FAERS identified significant disproportionality signals, particularly for IL-12/23 and IL-23 inhibitors. This discrepancy may be attributed to differences in study design, as randomized controlled trials are often limited by relatively short follow-up durations, strict inclusion criteria, and insufficient power to detect rare or long-latency adverse events such as malignancies (Hong et al., 2021). Conversely, spontaneous reporting systems are more sensitive to rare safety signals in real-world settings, albeit at the expense of potential reporting biases and lack of causality assessment. Therefore, our findings should be interpreted as hypothesis-generating signals that warrant further validation in large-scale, long-term clinical studies.

Several limitations of this study should be acknowledged. First, as a spontaneous reporting system, FAERS is inherently subject to underreporting, reporting bias, and stimulated reporting, which may distort the true incidence of adverse events. Second, the lack of denominator data precludes the calculation of incidence rates and limits causal inference between interleukin inhibitors and skin cancer. Third, important clinical information, such as treatment duration, cumulative dose, comorbidities, phototherapy (lacks standardized drug codes in FAERS), and prior history of skin cancer, is unavailable or incomplete in FAERS, preventing adequate adjustment for potential confounders. Consequently, our subgroup findings are exploratory and should not be interpreted as adjusted risk estimates**.** Fourth, inherent market time bias must be acknowledged when interpreting the safety profiles. Ustekinumab, approved earliest in 2009, benefits from over a decade of real-world clinical exposure, naturally increasing its cumulative report volume. Conversely, newer generation biologics, particularly the dual IL-17A/F inhibitor bimekizumab approved in 2023, have limited post-marketing exposure years and a smaller patient denominator within the FAERS database. Furthermore, while standard thresholds used in this study minimize false positives across multiple comparisons, they are conservative for low-frequency agents like bimekizumab. In such cases, limited reports widen confidence intervals, potentially masking early safety signals. Therefore, the lack of a disproportionality signal for bimekizumab and the weaker signals for newer agents likely reflect their shorter lifespan on the market and the high conservatism of standard algorithms, rather than a definitive guarantee of superior oncogenic safety. Fifth, the high missing data rate for age is an inherent limitation of FAERS that may introduce selection bias and reduce the statistical power of our age-stratified subgroup analyses. Because demographic fields are non-mandatory, incomplete reporting is common. While this missingness requires cautious interpretation, the stratified findings remain valuable as exploratory signals rather than definitive epidemiological estimates. In addition, duplicate reports and variable data quality may further affect the robustness of the analysis, although standard deduplication procedures were applied. Finally, disproportionality analyses are primarily hypothesis-generating and cannot establish causality; therefore, our findings should be interpreted with caution and validated in well-designed prospective studies.

## Conclusion

This pharmacovigilance study using the FAERS database revealed disproportionate reporting of skin cancer associated with IL-12/23, IL-23, and IL-17 inhibitors, with the strongest signals observed for risankizumab and ustekinumab. In psoriasis-restricted sensitivity analyses, positive signals for IL-12/23 and IL-23 inhibitors remained consistent, whereas those for IL-17 inhibitors were no longer significant. The association was more pronounced in elderly patients, and temporal patterns suggested a statistically increasing reporting rate over time for certain agents. These findings underscore the importance of dermatological surveillance in patients receiving these biologics, particularly among older adults (aged >75 years) and those on long-term therapy. However, clinical management recommendations cannot be made based on these findings alone due to the inherent limitations of pharmacovigilance studies. Further validation through large-scale, long-term prospective studies is warranted to clarify the risk-benefit profile of individual interleukin inhibitors.

## Data Availability

The original contributions presented in the study are included in the article/Supplementary Material, further inquiries can be directed to the corresponding author.

## References

[B1] Ahmed A. Sonnenberg G. F. (2026). Interleukin-23 biology linking mucosal immunity to autoimmune diseases and cancer. Trends Immunol. 47, 397–408. 10.1016/j.it.2025.12.005 41483965

[B2] Arana A. Pottegård A. Kuiper J. G. Booth H. Reutfors J. Calingaert B. (2021). Long-Term risk of skin cancer and lymphoma in users of topical tacrolimus and pimecrolimus: final results from the extension of the cohort Study protopic joint European longitudinal lymphoma and skin cancer evaluation (JOELLE). Clin. Epidemiol. 13, 1141–1153. 10.2147/CLEP.S331287 35002327 PMC8721027

[B3] Asgari M. M. Ray G. T. Geier J. L. Quesenberry C. P. (2017). Malignancy rates in a large cohort of patients with systemically treated psoriasis in a managed care population. J. Am. Acad. Dermatol 76, 632–638. 10.1016/j.jaad.2016.10.006 28162854

[B4] Bencardino S. Bernardi F. Allocca M. Zilli A. Furfaro F. Peyrin-Biroulet L. (2025). Advanced therapies for inflammatory bowel disease and risk of skin cancer: what’s new? Cancers (Basel) 17, 1710. 10.3390/cancers17101710 40427207 PMC12110305

[B5] Bray F. Laversanne M. Sung H. Ferlay J. Siegel R. L. Soerjomataram I. (2024). Global cancer statistics 2022: GLOBOCAN estimates of incidence and mortality worldwide for 36 cancers in 185 countries. CA Cancer J. Clin. 74, 229–263. 10.3322/caac.21834 38572751

[B6] Dunn G. P. Koebel C. M. Schreiber R. D. (2006). Interferons, immunity and cancer immunoediting. Nat. Rev. Immunol. 6, 836–848. 10.1038/nri1961 17063185

[B7] Esse S. Mason K. J. Green A. C. Warren R. B. (2020). Melanoma risk in patients treated with biologic therapy for common inflammatory diseases: a systematic review and meta-analysis. JAMA Dermatol 156, 787–794. 10.1001/jamadermatol.2020.1300 32432649 PMC7240639

[B8] Fusaroli M. Salvo F. Begaud B. AlShammari T. M. Bate A. Battini V. (2024). The reporting of a disproportionality analysis for drug safety signal detection using individual case safety reports in PharmacoVigilance (READUS-PV): development and statement. Drug Saf. 47, 575–584. 10.1007/s40264-024-01421-9 38713346 PMC11116242

[B9] Garritsen F. M. van der Schaft J. van den Reek J. M. Politiek K. van Os-Medendorp H. van Dijk M. (2017). Risk of non-melanoma skin cancer in patients with Atopic dermatitis treated with oral immunosuppressive drugs. Acta Derm. Venereol. 97, 724–730. 10.2340/00015555-2637 28218345

[B10] Geller S. Xu H. Lebwohl M. Nardone B. Lacouture M. E. Kheterpal M. (2018). Malignancy risk and recurrence with psoriasis and its treatments: a concise update. Am. J. Clin. Dermatol 19, 363–375. 10.1007/s40257-017-0337-2 29260411 PMC5948118

[B11] Gibson M. Allen V. Adas M. Muzafar I. Muzafar S. Suresh N. (2026). Cancer risk of Janus kinase inhibitors and other advanced therapies in immune-mediated inflammatory diseases: a systematic review and Bayesian network meta-analysis of RCTs. Ann. Rheum. Dis. S0003-4967 (26), 00366–3. 10.1016/j.ard.2026.06.017 42431784

[B12] Hashizume H. Nakatani E. Sasaki H. Miyachi Y. (2023). Hydrochlorothiazide increases risk of nonmelanoma skin cancer in an elderly Japanese cohort with hypertension: the Shizuoka study. JAAD Int. 12, 49–57. 10.1016/j.jdin.2023.04.007 37274382 PMC10236168

[B13] Hong H. Wang C. Rosner G. L. (2021). Meta-analysis of rare adverse events in randomized clinical trials: bayesian and frequentist methods. Clin. Trials 18, 3–16. 10.1177/1740774520969136 33258698 PMC8041270

[B14] Jin B. Zhang Y. Miller H. D. He L. Ge D. Wang A. R. (2022). Defect of IL17 signaling, but not centrinone, inhibits the development of psoriasis and skin papilloma in mouse models. Biomedicines 10, 1976. 10.3390/biomedicines10081976 36009523 PMC9405709

[B15] Jiyad Z. Olsen C. M. Burke M. T. Isbel N. M. Green A. C. (2016). Azathioprine and risk of skin cancer in organ transplant recipients: systematic review and meta-analysis. Am. J. Transpl. 16, 3490–3503. 10.1111/ajt.13863 27163483

[B16] Kharouf F. Gladman D. D. (2024). Advances in the management of psoriatic arthritis in adults. BMJ 387, e081860. 10.1136/bmj-2024-081860 39572047

[B17] Kreher M. A. Konda S. Noland M. M. B. Longo M. I. Valdes-Rodriguez R. (2023a). Risk of melanoma and nonmelanoma skin cancer with immunosuppressants, part II: methotrexate, alkylating agents, biologics, and small molecule inhibitors. J. Am. Acad. Dermatol 88, 534–542. 10.1016/j.jaad.2022.11.043 36460256

[B18] Kreher M. A. Noland M. M. B. Konda S. Longo M. I. Valdes-Rodriguez R. (2023b). Risk of melanoma and nonmelanoma skin cancer with immunosuppressants, part I: calcineurin inhibitors, thiopurines, IMDH inhibitors, mTOR inhibitors, and corticosteroids. J. Am. Acad. Dermatol 88, 521–530. 10.1016/j.jaad.2022.11.044 36460257

[B19] Krzysztofik M. Brzewski P. Cuber P. Kacprzyk A. Kulbat A. Richter K. (2023). Risk of Melanoma and Non-Melanoma skin cancer in patients with psoriasis and psoriatic arthritis treated with targeted therapies: a systematic review and meta-analysis. Pharm. (Basel) 17, 14. 10.3390/ph17010014 38276003 PMC10820691

[B20] Langowski J. L. Zhang X. Wu L. Mattson J. D. Chen T. Smith K. (2006). IL-23 promotes tumour incidence and growth. Nature 442, 461–465. 10.1038/nature04808 16688182

[B21] Leonardi C. Papp K. Strober B. Thaçi D. Warren R. B. Tyring S. (2019). Comprehensive long-term safety of adalimumab from 18 clinical trials in adult patients with moderate-to-severe plaque psoriasis. Br. J. Dermatol 180, 76–85. 10.1111/bjd.17084 30169904

[B22] Linares M. A. Zakaria A. Nizran P. (2015). Skin cancer. Prim. Care 42, 645–659. 10.1016/j.pop.2015.07.006 26612377

[B23] Liu R. Wan Q. Zhao R. Xiao H. Cen Y. Xu X. (2021). Risk of non-melanoma skin cancer with biological therapy in common inflammatory diseases: a systemic review and meta-analysis. Cancer Cell Int. 21, 614. 10.1186/s12935-021-02325-9 34809619 PMC8607648

[B24] Liu Z. Liang Q. Ren Y. Guo C. Ge X. Wang L. (2023). Immunosenescence: molecular mechanisms and diseases. Signal Transduct. Target Ther. 8, 200. 10.1038/s41392-023-01451-2 37179335 PMC10182360

[B25] Newton-Bishop J. Bishop D. T. Harland M. (2020). Melanoma genomics. Acta Derm. Venereol. 100, adv00138. 10.2340/00015555-3493 32346746 PMC9189746

[B26] Potestio L. Tommasino N. Lauletta G. Salsano A. Lucagnano G. Menna L. (2024). The impact of psoriasis treatments on the risk of skin cancer: a narrative review. Adv. Ther. 41, 3778–3791. 10.1007/s12325-024-02968-w 39196500

[B27] Sauzet O. Cornelius V. (2022). Generalised weibull model-based approaches to detect non-constant hazard to signal adverse drug reactions in longitudinal data. Front. Pharmacol. 13, 889088. 10.3389/fphar.2022.889088 36081935 PMC9445551

[B28] Scott F. I. Mamtani R. Brensinger C. M. Haynes K. Chiesa-Fuxench Z. C. Zhang J. (2016). Risk of nonmelanoma skin cancer associated with the use of immunosuppressant and biologic agents in patients with a history of autoimmune disease and nonmelanoma skin cancer. JAMA Dermatol 152, 164–172. 10.1001/jamadermatol.2015.3029 26510126 PMC5935268

[B29] Semaka A. Salopek T. G. (2022). Risk of developing melanoma with systemic agents used to treat psoriasis: a review of the literature. J. Cutan. Med. Surg. 26, 87–92. 10.1177/12034754211038509 34392725 PMC8750137

[B30] Smith S. D. Stratigos A. Augustin M. Carrascosa J. M. Grond S. Riedl E. (2023). Integrated safety analysis on skin cancers among patients with psoriasis receiving ixekizumab in clinical trials. Dermatol Ther. (Heidelb) 13, 1773–1787. 10.1007/s13555-023-00966-4 37351831 PMC10366039

[B31] Stern R. S. , and PUVA Follow-Up Study (2012). The risk of squamous cell and basal cell cancer associated with psoralen and ultraviolet A therapy: a 30-year prospective study. J. Am. Acad. Dermatol 66, 553–562. 10.1016/j.jaad.2011.04.004 22264671

[B32] Teng M. W. L. Andrews D. M. McLaughlin N. von Scheidt B. Ngiow S. F. Möller A. (2010). IL-23 suppresses innate immune response independently of IL-17A during carcinogenesis and metastasis. Proc. Natl. Acad. Sci. U. S. A. 107, 8328–8333. 10.1073/pnas.1003251107 20404142 PMC2889517

[B33] Teng M. W. L. Bowman E. P. McElwee J. J. Smyth M. J. Casanova J.-L. Cooper A. M. (2015). IL-12 and IL-23 cytokines: from discovery to targeted therapies for immune-mediated inflammatory diseases. Nat. Med. 21, 719–729. 10.1038/nm.3895 26121196

[B34] Wang R. Chen Y. Shao X. Chen T. Zhong J. Ou Y. (2025). Burden of skin Cancer in older adults from 1990 to 2021 and modelled Projection to 2050. JAMA Dermatol 161, 715–722. 10.1001/jamadermatol.2025.1276 40397469 PMC12096324

[B35] Weiss-Smith S. Deshpande G. Chung S. Gogolak V. (2011). The FDA drug safety surveillance program: adverse event reporting trends. Arch. Intern Med. 171, 591–593. 10.1001/archinternmed.2011.89 21444854

[B36] Wu S. Xu Y. Yang L. Guo L. Jiang X. (2023). Short-term risk and long-term incidence rate of infection and malignancy with IL-17 and IL-23 inhibitors in adult patients with psoriasis and psoriatic arthritis: a systematic review and meta-analysis. Front. Immunol. 14, 1294416. 10.3389/fimmu.2023.1294416 38106423 PMC10721978

[B37] Yu S.-Y. Hirsch A. Zaslavsky O. Cochrane B. B. (2023). Risk factors and early prevention of skin cancer in rural older outdoor workers: a scoping review. Geriatr. Nurs. 54, 37–45. 10.1016/j.gerinurse.2023.08.017 37703688

[B38] Zhao H. Wu L. Yan G. Chen Y. Zhou M. Wu Y. (2021). Inflammation and tumor progression: signaling pathways and targeted intervention. Signal Transduct. Target Ther. 6, 263. 10.1038/s41392-021-00658-5 34248142 PMC8273155

