# Supplementary material for: Potential association between IL-12/23, IL-23, and IL-17 inhibitors and skin cancer: a pharmacovigilance study based on the FAERS database
Source: Front Pharmacol. 2026 Oct 22;17:1944368. doi: 10.3389/fphar.2026.1944368 (PMC13736627; doi:10.3389/fphar.2026.1944368)
Supplement: Supplementary file 1 [file Table1.docx]

Potential association between IL-12/23, IL-23, and IL-17 inhibitors and skin cancer: a pharmacovigilance study based on the FAERS database

Supplementary Table S1. Complete List of MedDRA Preferred Terms for Skin Malignant Neoplasms

Supplementary Table S2. Safety signals for the interleukin inhibitors

Supplementary Table S3. Safety signals for interleukin inhibitors (sensitivity analysis excluding non-specific PTs from the SMQ "Skin Malignant Neoplasms")

Supplementary Table S4. Safety signals for interleukin inhibitors in a sensitivity analysis restricted to psoriasis/psoriatic arthritis

Supplementary Table S5. Safety signals for interleukin inhibitors in a sensitivity analysis restricted to healthcare professional reports

Supplementary Table S6. Subgroup analysis of skin cancer-related adverse events with interleukin inhibitors

Supplementary Table S7. Disproportionality analysis of top 10 adverse event PTs associated with interleukin inhibitors

Supplementary Table S8. Skin cancer signals for TNF inhibitors, JAK inhibitors, and apremilast in patients with psoriasis and psoriatic arthritis.

Supplementary Table S9. Calendar period-stratified analysis of skin cancer signals for interleukin inhibitors with sufficient reports

Supplementary Table S10. Weibull time-to-onset analysis results for interleukin inhibitors

Supplementary Table S11. Co-medication analysis of interleukin inhibitors with concomitant drugs associated with skin cancer

Supplementary Table S12. READUS-PV checklist

Supplementary Table S1. Complete List of MedDRA Preferred Terms for Skin Malignant Neoplasms

"MYCOSIS FUNGOIDES",

"LENTIGO MALIGNA STAGE UNSPECIFIED",

"DERMATOFIBROSARCOMA",

"PENIS CARCINOMA",

"ACRAL LENTIGINOUS MELANOMA STAGE UNSPECIFIED",

"PENILE MALIGNANT NEOPLASM",

"PAGET'S DISEASE OF SKIN",

"MYCOSIS FUNGOIDES STAGE IV",

"LYMPHOMA CUTIS",

"MYCOSIS FUNGOIDES RECURRENT",

"MYCOSIS FUNGOIDES STAGE I",

"MYCOSIS FUNGOIDES STAGE II",

"MYCOSIS FUNGOIDES REFRACTORY",

"ANAPLASTIC LARGE-CELL LYMPHOMA",

"MYCOSIS FUNGOIDES STAGE III",

"SPITZOID MELANOMA",

"VULVECTOMY",

"VULVAR ADENOCARCINOMA",

"VULVAL CANCER STAGE IV",

"VULVAL CANCER STAGE III",

"VULVAL CANCER STAGE II",

"VULVAL CANCER STAGE I",

"VULVAL CANCER STAGE 0",

"VULVAL CANCER RECURRENT",

"VULVAL CANCER METASTATIC",

"VULVAL CANCER",

"TRICHOBLASTIC CARCINOMA",

"SUPERFICIAL SPREADING MELANOMA STAGE UNSPECIFIED",

"SUPERFICIAL SPREADING MELANOMA STAGE IV",

"SUPERFICIAL SPREADING MELANOMA STAGE III",

"SUPERFICIAL SPREADING MELANOMA STAGE II",

"SUPERFICIAL SPREADING MELANOMA STAGE I",

"SQUAMOUS CELL CARCINOMA OF THE VULVA",

"SQUAMOUS CELL CARCINOMA OF SKIN",

"SQUAMOUS CELL CARCINOMA",

"SQUAMOPROLIFERATIVE LESION",

"SKIN SQUAMOUS CELL CARCINOMA RECURRENT",

"SKIN SQUAMOUS CELL CARCINOMA METASTATIC",

"SKIN CANCER METASTATIC",

"SKIN CANCER",

"SKIN ANGIOSARCOMA",

"SEBACEOUS CARCINOMA",

"SARCOMA OF SKIN",

"QUEYRAT ERYTHROPLASIA",

"POROCARCINOMA",

"PILOMATRIX CARCINOMA",

"PERIPHERAL T-CELL LYMPHOMA UNSPECIFIED STAGE IV",

"PERIPHERAL T-CELL LYMPHOMA UNSPECIFIED STAGE III",

"PERIPHERAL T-CELL LYMPHOMA UNSPECIFIED STAGE II",

"PERIPHERAL T-CELL LYMPHOMA UNSPECIFIED STAGE I",

"PERIPHERAL T-CELL LYMPHOMA UNSPECIFIED REFRACTORY",

"PERIPHERAL T-CELL LYMPHOMA UNSPECIFIED RECURRENT",

"PERIPHERAL T-CELL LYMPHOMA UNSPECIFIED",

"PENIS CARCINOMA STAGE IV",

"PENIS CARCINOMA STAGE II",

"PENIS CARCINOMA RECURRENT",

"PENIS CARCINOMA METASTATIC",

"PENILE SQUAMOUS CELL CARCINOMA",

"PENILE CANCER",

"PAGET'S DISEASE OF PENIS",

"NODULAR MELANOMA",

"NEUROENDOCRINE CARCINOMA OF THE SKIN",

"NAEVOID MELANOMA",

"METASTATIC SQUAMOUS CELL CARCINOMA",

"METASTATIC MALIGNANT MELANOMA",

"MELANOMA RECURRENT",

"MARJOLIN'S ULCER",

"MALIGNANT SWEAT GLAND NEOPLASM",

"MALIGNANT MELANOMA STAGE IV",

"MALIGNANT MELANOMA STAGE III",

"MALIGNANT MELANOMA STAGE II",

"MALIGNANT MELANOMA STAGE I",

"MALIGNANT MELANOMA OF EYELID",

"MALIGNANT MELANOMA IN SITU",

"MALIGNANT MELANOMA",

"MALIGNANT BLUE NAEVUS",

"LOCALISED MELANOMA",

"LENTIGO MALIGNA STAGE III",

"LENTIGO MALIGNA STAGE II",

"LENTIGO MALIGNA STAGE I",

"LENTIGO MALIGNA",

"KERATOACANTHOMA",

"KAPOSI'S SARCOMA CLASSICAL TYPE",

"KAPOSI'S SARCOMA AIDS RELATED",

"KAPOSI'S SARCOMA",

"IATROGENIC KAPOSI SARCOMA",

"HIDRADENOCARCINOMA",

"EXTRAMAMMARY PAGET'S DISEASE",

"ELECTRON RADIATION THERAPY TO SKIN",

"ECCRINE CARCINOMA",

"DESMOPLASTIC MELANOMA",

"DERMATOFIBROSARCOMA PROTUBERANS METASTATIC",

"DERMATOFIBROSARCOMA PROTUBERANS",

"CUTANEOUS T-CELL LYMPHOMA STAGE IV",

"CUTANEOUS T-CELL LYMPHOMA STAGE III",

"CUTANEOUS T-CELL LYMPHOMA STAGE II",

"CUTANEOUS T-CELL LYMPHOMA STAGE I",

"CUTANEOUS T-CELL LYMPHOMA REFRACTORY",

"CUTANEOUS T-CELL LYMPHOMA RECURRENT",

"CUTANEOUS T-CELL LYMPHOMA",

"CUTANEOUS LYMPHOMA",

"CARCINOMA IN SITU OF SKIN",

"CARCINOMA IN SITU OF PENIS",

"BOWEN'S DISEASE",

"BLASTIC PLASMACYTOID DENDRITIC CELL NEOPLASIA",

"BASOSQUAMOUS CARCINOMA OF SKIN",

"BASOSQUAMOUS CARCINOMA",

"BASAL CELL CARCINOMA METASTATIC",

"BASAL CELL CARCINOMA",

"ATYPICAL FIBROXANTHOMA",

"ANAPLASTIC LARGE CELL LYMPHOMA T- AND NULL-CELL TYPES",

"ACRAL LENTIGINOUS MELANOMA STAGE IV",

"ACRAL LENTIGINOUS MELANOMA STAGE III",

"ACRAL LENTIGINOUS MELANOMA STAGE II",

"ACRAL LENTIGINOUS MELANOMA STAGE I",

"ACRAL LENTIGINOUS MELANOMA",

"BLASTIC PLASMACYTOID DENDRITIC CELL NEOPLASIA"

| Supplementary Table S2. Safety signals for the interleukin inhibitors | | | | |  |
| --- | --- | --- | --- | --- | --- |
| **Drug** | **N** | **ROR (95% CI)** | **IC_025_** | **P-value** | **Signal** |
| Drugs of interest | 3661 | 3.31 (3.20-3.42) | 1.62 | <0.001 | Yes |
| Ustekinumab | 1182 | 5.53 (5.23-5.85) | 2.35 | <0.001 | Yes |
| Guselkumab | 107 | 1.57 (1.31-1.89) | 0.37 | <0.001 | Yes |
| Risankizumab | 1525 | 8.01 (7.62-8.42) | 2.88 | <0.001 | Yes |
| Tildrakizumab | 33 | 5.81 (4.13-8.19) | 1.84 | <0.001 | Yes |
| Secukinumab | 639 | 1.18 (1.09-1.27) | 0.12 | <0.001 | Yes |
| Ixekizumab | 134 | 1.72 (1.45-2.03) | 0.53 | <0.001 | Yes |
| Brodalumab | 21 | 2.31 (1.53-3.47) | 0.54 | <0.001 | Yes |
| Bimekizumab | 20 | 0.80 (0.51-1.24) | -0.94 | 0.718 | No |

P-values are Bonferroni-corrected. N, number of reports with both the drugs and skin cancer; ROR, reporting odds ratios; CI, confidence interval; IC_025_, the lower bound of the 95% credibility interval for the Information Component.

Supplementary Table S3. Safety signals for interleukin inhibitors (sensitivity analysis excluding non-specific PTs from the SMQ "Skin Malignant Neoplasms")

| **Drug** | **N** | **ROR (95% CI)** | **IC_025_** | **P-value** | **Signal** |
| --- | --- | --- | --- | --- | --- |
| Drugs of interest | 3463 | 3.53 (3.41-3.65) | 1.71 | <0.001 | Yes |
| Ustekinumab | 1142 | 5.87 (5.54-6.22) | 2.43 | <0.001 | Yes |
| Guselkumab | 101 | 1.62 (1.34-1.96) | 0.41 | <0.001 | Yes |
| Risankizumab | 1429 | 8.48 (8.05-8.93) | 2.96 | <0.001 | Yes |
| Tildrakizumab | 26 | 5.40 (3.67-7.95) | 1.65 | <0.001 | Yes |
| Secukinumab | 597 | 1.23 (1.14-1.33) | 0.18 | <0.001 | Yes |
| Ixekizumab | 129 | 1.81 (1.53-2.15) | 0.60 | <0.001 | Yes |
| Brodalumab | 21 | 2.72 (1.81-4.10) | 0.75 | <0.001 | Yes |
| Bimekizumab | 18 | 1.38 (0.87-2.19) | -0.23 | 0.443 | No |
| Note: Of the 117 PTs in the SMQ "Skin Malignant Neoplasms" (Broad), 29 non-specific PTs (e.g., “VULVECTOMY”, “VULVAL CANCER” and “PENILE CANCER”) were excluded to restrict the analysis to definitive skin cancer cases. P-values are Bonferroni-corrected. N, number of reports with both the drugs and skin cancer; ROR, reporting odds ratios; CI, confidence interval; IC_025_, the lower bound of the 95% credibility interval for the Information Component. | | | | | |

Supplementary Table S4. Safety signals for interleukin inhibitors in a sensitivity analysis restricted to psoriasis/psoriatic arthritis

| **Drug** | **N** | **ROR (95% CI)** | **IC_025_** | **P-value** | **Signal** |
| --- | --- | --- | --- | --- | --- |
| Drugs of interest | 2747 | 2.00 (1.91-2.10) | 0.6 | <0.001 | Yes |
| Ustekinumab | 807 | 4.37 (4.06-4.70) | 1.86 | <0.001 | Yes |
| Guselkumab | 80 | 0.78 (0.62-0.97) | -0.68 | 0.055 | No |
| Risankizumab | 1240 | 4.41 (4.15-4.70) | 1.81 | <0.001 | Yes |
| Tildrakizumab | 22 | 2.58 (1.70-3.93) | 0.66 | <0.001 | Yes |
| Secukinumab | 463 | 0.47 (0.43-0.52) | -1.11 | <0.001 | No |
| Ixekizumab | 100 | 0.79 (0.64-0.96) | -0.63 | 0.037 | No |
| Brodalumab | 20 | 0.96 (0.62-1.49) | -0.69 | 1.000 | No |
| Bimekizumab | 15 | 0.51 (0.31-0.85) | -1.64 | 0.022 | No |
| P-values are Bonferroni-corrected. N, number of reports with both the drugs and skin cancer; ROR, reporting odds ratios; CI, confidence interval; IC_025_, the lower bound of the 95% credibility interval for the Information Component. | | | | | |

Supplementary Table S5. Safety signals for interleukin inhibitors in a sensitivity analysis restricted to healthcare professional reports

| **Drug** | **N** | **ROR (95% CI)** | **IC_025_** | **P-value** | **Signal** |
| --- | --- | --- | --- | --- | --- |
| Drugs of interest | 1282 | 3.26 (3.11-3.42) | 1.59 | <0.001 | Yes |
| Ustekinumab | 596 | 5.92 (5.55-6.32) | 2.43 | <0.001 | Yes |
| Guselkumab | 87 | 1.93 (1.58-2.36) | 0.63 | <0.001 | Yes |
| Risankizumab | 147 | 5.03 (4.29-5.90) | 2.05 | <0.001 | Yes |
| Tildrakizumab | 22 | 7.71 (5.06-11.74) | 1.96 | <0.001 | Yes |
| Secukinumab | 355 | 1.67 (1.52-1.84) | 0.59 | <0.001 | Yes |
| Ixekizumab | 50 | 2.01 (1.56-2.58) | 0.62 | <0.001 | Yes |
| Brodalumab | 15 | 5.80 (3.69-9.11) | 1.57 | <0.001 | Yes |
| Bimekizumab | 10 | 1.27(0.72-2.24) | -0.48 | 0.99 | No |
| Reports from healthcare professionals only (physicians, pharmacists, nurses, and other health professionals). Consumer/patient self-reports were excluded. P-values are Bonferroni-corrected. N, number of reports with both the drugs and skin cancer; ROR, reporting odds ratios; CI, confidence interval; IC_025_, the lower bound of the 95% credibility interval for the Information Component. | | | | | |

Supplementary Table S6. Subgroup analysis of skin cancer-related adverse events with interleukin inhibitors

| Subgroup | Ustekinumab | Guselkumab | Risankizumab | Tildrakizumab | Secukinumab | Ixekizumab | Brodalumab | Bimekizumab |
| --- | --- | --- | --- | --- | --- | --- | --- | --- |
| Age |  |  |  |  |  |  |  |  |
| <18 years | **34,**  **12.23 (8.68-17.24),**  **2.67** | 1,  2.71 (0.38-19.25), -1.5 | NA | NA | 1,  2.45 (0.34-17.45), -1.54 | NA | NA | 1,  39.3 (5.45-283.43),  -1.11 |
| 18-65 years | **412,**  **5.05 (4.59-5.55), 2.15** | 27,  0.98 (0.67-1.43), -0.57 | **334,**  **6.21 (5.59-6.9),**  **2.43** | **11,**  **9.58 (5.29-17.35), 1.63** | 169,  0.87 (0.75-1),  -0.42 | **69,**  **2.24 (1.78-2.83),**  **0.80** | **12,**  **2.81 (1.66-4.74),**  **0.57** | 3,  0.48 (0.16-1.5),  -2.29 |
| 66-75 years | **227,**  **9.84 (8.66-11.18),**  **3.01** | **16,**  **2.35 (1.48-3.74),**  **0.47** | **223,**  **7.32 (6.43-8.33),**  **2.61** | **7,**  **6.40(3.04-13.49),**  **0.9** | **83,**  **1.62(1.32-1.99),**  **0.38** | **18,**  **2.62(1.70-4.02),**  **0.66** | 3,  1.18(0.38-3.67),  -1.27 | 2,  1.46 (0.36-5.85),  -1.33 |
| >75 years | **93,**  **11.4 (9.45-13.75),**  **3.07** | **11,**  **3.47 (1.92-6.27),**  **0.68** | **143,**  **9.81 (8.34-11.55)**  **2.93** | **4,**  **8.68 (3.23-23.3),**  **0.46** | **24,**  **1.79 (1.25-2.54),**  **0.29** | **7,**  **3.87 (2.08-7.20),**  **0.74** | 4,  2.44 (0.91-6.51),  -0.38 | 1,  2.84 (0.40-20.24),  -1.49 |
| Sex |  |  |  |  |  |  |  |  |
| Female | **508,**  **5.93 (5.44-6.47), 2.4** | 33,  1.19 (0.84-1.67), -0.26 | **737,**  **8.97 (8.35-9.64),**  **3.00** | **14,**  **6.83 (4.04-11.55), 1.54** | **302,**  **1.18 (1.06-1.32), 0.08** | **62,**  **1.86 (1.46-2.37),**  **0.52** | **9,**  **2.8 (1.51-5.21), 0.39** | 12,  1.06 (0.6-1.87),  -0.72 |
| Male | **562,**  **5.16 (4.76-5.6), 2.21** | **46,**  **1.39 (1.05-1.85), 0.05** | **756,**  **7.4 (6.9-7.95),**  **2.73** | **19,**  **5.61(3.57-8.82),**  **1.53** | **292,**  **1.15 (1.03-1.29), 0.04** | **62,**  **1.61(1.26-2.07),**  **0.31** | **12,**  **1.84(1.07-3.17), 0.02** | 6,  0.49 (0.22-1.08),  -2.02 |
| Bold values indicate statistically significant positive signals (defined as N≥3, ROR 95% CI > 1 and IC_025_ > 0). Values in each cell were presented as number of cases (N), reporting odds ratio (95% confidence interval, 95%CI) and lower bound of information component (IC₀₂₅) in sequence. NA, not available. | | | | | | | | |

Supplementary Table S7. Disproportionality analysis of top 10 adverse event PTs associated with interleukin inhibitors

| PTs | Durgs | N | ROR (95% CI) | IC(IC025) | P-value | Signal |
| --- | --- | --- | --- | --- | --- | --- |
| Skin Cancer | Ustekinumab | 223 | 3.84 (3.36-4.38) | 1.93 (1.71) | <0.001 | Yes |
|  | Guselkumab | 21 | 1.13 (0.73-1.73) | 0.17 (-0.45) | 1.000 | No |
|  | Risankizumab | 749 | 14.95 (13.9-16.09) | 3.84 (3.71) | <0.001 | Yes |
|  | Tildrakizumab | 2 | 1.36 (0.34-5.45) | 0.45 (-1.38) | 1.000 | No |
|  | Secukinumab | 151 | 1.02 (0.87-1.2) | 0.03 (-0.21) | 1.000 | No |
|  | Ixekizumab | 19 | 0.92 (0.59-1.44) | -0.12 ( -0.76 ) | 1.000 | No |
|  | Brodalumab | 7 | 2.73 (1.3-5.73) | 1.45 (0.14) | 0.145 | Yes |
|  | Bimekizumab | 9 | 1.4 (0.73-2.69) | 0.48 (-0.49) | 1.000 | No |
| Basal Cell Carcinoma | Ustekinumab | 347 | 8.29 (7.45-9.23) | 3.02 (2.83) | <0.001 | Yes |
|  | Guselkumab | 21 | 1.54 (1.01-2.37) | 0.62 (-0.03) | 1.000 | No |
|  | Risankizumab | 304 | 8.15 (7.27-9.13) | 3 (2.8) | <0.001 | Yes |
|  | Tildrakizumab | 8 | 7.49 (3.74-14.98) | 2.9 (1.16) | <0.001 | Yes |
|  | Secukinumab | 144 | 1.34 (1.13-1.58) | 0.41 (0.17) | 0.022 | Yes |
|  | Ixekizumab | 20 | 1.33 (0.86-2.06) | 0.41 (-0.24) | 1.000 | No |
|  | Brodalumab | 5 | 2.67 (1.11-6.42) | 1.42 (-0.12) | 0.377 | No |
|  | Bimekizumab | NA | NA | NA | NA | NA |
| Malignant Melanoma | Ustekinumab | 207 | 5.68 (4.95-6.52) | 2.49 (2.25) | <0.001 | Yes |
|  | Guselkumab | 26 | 2.21 (1.51 - 3.25) | 1.14 (0.53) | 0.001 | Yes |
|  | Risankizumab | 199 | 6.14 (5.34-7.07) | 2.6 (2.36) | <0.001 | Yes |
|  | Tildrakizumab | 8 | 8.67 (4.33-17.36) | 3.11 (1.26) | <0.001 | Yes |
|  | Secukinumab | 158 | 1.7 (1.46-1.99) | 0.76 (0.52) | <0.001 | Yes |
|  | Ixekizumab | 47 | 3.63 (2.72-4.83) | 1.85 (1.36) | <0.001 | Yes |
|  | Brodalumab | 2 | 1.24 (0.31-4.95) | 0.31 (-1.47) | 1.000 | No |
|  | Bimekizumab | 6 | 1.48 (0.66-3.29) | 0.56 (-0.62) | 1.000 | No |
| Squamous Cell Carcinoma | Ustekinumab | 243 | 9.69 (8.53-11.01) | 3.24 (3) | <0.001 | Yes |
|  | Guselkumab | 17 | 2.08 (1.29-3.34) | 1.05 (0.29) | 0.067 | Yes |
|  | Risankizumab | 117 | 5.17 (4.3-6.2) | 2.35 (2.04) | <0.001 | Yes |
|  | Tildrakizumab | 5 | 7.77 (3.23-18.69) | 2.96 (0.69) | 0.005 | Yes |
|  | Secukinumab | 71 | 1.09 (0.87-1.38) | 0.13 (-0.22) | 1.000 | No |
|  | Ixekizumab | 5 | 0.55 (0.23-1.32) | -0.86 (-1.93) | 1.000 | No |
|  | Brodalumab | 3 | 2.66 (0.86-8.26) | 1.41 (-0.53) | 0.945 | No |
|  | Bimekizumab | 2 | 0.71 (0.18-2.83) | -0.5 (-2.02) | 1.000 | No |
| Squamous Cell Carcinoma Of Skin | Ustekinumab | 55 | 3.38 (2.59-4.41) | 1.75 (1.3) | <0.001 | Yes |
|  | Guselkumab | 8 | 1.54 (0.77-3.07) | 0.62 (-0.43) | 1.000 | No |
|  | Risankizumab | 111 | 7.76 (6.43-9.36) | 2.93 (2.57) | <0.001 | Yes |
|  | Tildrakizumab | 6 | 14.67 (6.58-32.69) | 3.87 (1.22) | <0.001 | Yes |
|  | Secukinumab | 42 | 1.02 (0.75-1.38) | 0.02 (-0.42) | 1.000 | No |
|  | Ixekizumab | 7 | 1.21 (0.58-2.55) | 0.28 (-0.78) | 1.000 | No |
|  | Brodalumab | 1 | 1.4 (0.2-9.91) | 0.48 (-1.82) | 1.000 | No |
|  | Bimekizumab | 2 | 1.11 (0.28-4.45) | 0.15 (-1.57) | 1.000 | No |
| Cutaneous T-Cell Lymphoma | Ustekinumab | 37 | 7.19 (5.19-9.96) | 2.82 (2.13) | <0.001 | Yes |
|  | Guselkumab | 6 | 3.61 (1.62-8.04) | 1.85 (0.3) | 0.131 | Yes |
|  | Risankizumab | 22 | 4.77 (3.13-7.26) | 2.24 (1.42) | <0.001 | Yes |
|  | Tildrakizumab | 1 | 7.64 (1.07-54.26) | 2.93 (-1.22) | 1.000 | No |
|  | Secukinumab | 32 | 2.45 (1.73-3.47) | 1.28 (0.71) | <0.001 | Yes |
|  | Ixekizumab | 11 | 6 (3.32-10.85) | 2.58 (1.24) | <0.001 | Yes |
|  | Brodalumab | 1 | 4.37 (0.61-31.02) | 2.13 (-1.34) | 1.000 | No |
|  | Bimekizumab | 1 | 1.74 (0.24-12.35) | 0.8 (-1.7) | 1.000 | No |
| Bowen's Disease | Ustekinumab | 19 | 5.65 (3.59-8.89) | 2.48 (1.53) | <0.001 | Yes |
|  | Guselkumab | NA | NA | NA | NA | NA |
|  | Risankizumab | 12 | 3.99 (2.26-7.05) | 1.99 (0.88) | 0.003 | Yes |
|  | Tildrakizumab | 1 | 11.74 (1.65-83.44) | 3.55 (-1.16) | 0.735 | No |
|  | Secukinumab | 15 | 1.75 (1.05-2.92) | 0.8 (0.01) | 1.000 | Yes |
|  | Ixekizumab | 2 | 1.67 (0.42-6.68) | 0.74 (-1.22) | 1.000 | No |
|  | Brodalumab | NA | NA | NA | NA | NA |
|  | Bimekizumab | NA | NA | NA | NA | NA |
| Keratoacanthoma | Ustekinumab | 14 | 7.63 (4.49-12.96) | 2.9 (1.63) | <0.001 | Yes |
|  | Guselkumab | 3 | 5.06 (1.63-15.73) | 2.33 (-0.12) | 0.407 | No |
|  | Risankizumab | 5 | 3.02 (1.25-7.28) | 1.59 (-0.01) | 0.951 | No |
|  | Tildrakizumab | NA | NA | NA | NA | NA |
|  | Secukinumab | 1 | 0.21 (0.03-1.5) | -2.24 (-3.56) | 1.000 | No |
|  | Ixekizumab | 3 | 4.57 (1.47-14.22) | 2.19 (-0.18) | 0.643 | No |
|  | Brodalumab | 1 | 12.24 (1.72-87.03) | 3.61 (-1.16) | 0.707 | No |
|  | Bimekizumab | NA | NA | NA | NA | NA |
| Metastatic Malignant Melanoma | Ustekinumab | 8 | 1.25 (0.62-2.5) | 0.32 (-0.68) | 1.000 | No |
|  | Guselkumab | 1 | 0.49 (0.07-3.48) | -1.03 (-2.65) | 1.000 | No |
|  | Risankizumab | 6 | 1.05 (0.47-2.35) | 0.07 (-1.03) | 1.000 | No |
|  | Tildrakizumab | NA | NA | NA | NA | NA |
|  | Secukinumab | 8 | 0.49 (0.25-0.99) | -1.02 (-1.9) | 1.000 | No |
|  | Ixekizumab | 3 | 1.33 (0.43-4.13) | 0.41 (-1.15) | 1.000 | No |
|  | Brodalumab | NA | NA | NA | NA | NA |
|  | Bimekizumab | NA | NA | NA | NA | NA |
| Cutaneous Lymphoma | Ustekinumab | 4 | 7.15 (2.66-19.25) | 2.81 (0.36) | 0.106 | Yes |
|  | Guselkumab | NA | NA | NA | NA | NA |
|  | Risankizumab | 5 | 10.11 (4.16-24.57) | 3.3 (0.8) | 0.006 | Yes |
|  | Tildrakizumab | NA | NA | NA | NA | NA |
|  | Secukinumab | 6 | 4.28 (1.9-9.64) | 2.06 (0.41) | 0.125 | Yes |
|  | Ixekizumab | 4 | 20.36 (7.56-54.8) | 4.32 (0.75) | 0.001 | Yes |
|  | Brodalumab | 2 | 81.14 (20.14-326.84) | 6.33 (-0.13) | 0.003 | No |
|  | Bimekizumab | NA | NA | NA | NA | NA |

P-values are Bonferroni-corrected. PT, preferred terms; NA, not available. N, number of reports with both the drugs and skin cancer; ROR, reporting odds ratios; CI, confidence interval; IC025, the lower bound of the 95% credibility interval for the Information Component.

| Supplementary Table S8. Skin cancer signals for TNF inhibitors, JAK inhibitors, and apremilast in patients with psoriasis and psoriatic arthritis. | | | | |
| --- | --- | --- | --- | --- |
| Drug | N | ROR (95% CI) | IC_025_ | Signal |
| JAKi (pooled) | 102 | 1.08 (0.88-1.31) | -0.18 | No |
| Tofacitinib | 42 | 0.67 (0.49-0.91) | -1.00 | No |
| Upadacitinib | 60 | 1.84 (1.42-2.37) | 0.47 | Yes |
| TNFi (pooled) | 2736 | 0.95 (0.9-0.99) | -0.11 | No |
| Infliximab | 599 | 3.97 (3.65-4.32) | 1.74 | Yes |
| Adalimumab | 1215 | 0.89 (0.84-0.95) | -0.22 | No |
| Etanercept | 745 | 0.62 (0.57-0.67) | -0.71 | No |
| Certolizumab pegol | 66 | 0.41 (0.32-0.52) | -1.62 | No |
| Golimumab | 111 | 2.44 (2.02-2.94 ) | 0.97 | Yes |
| Apremilast (PDE4i) | 281 | 0.38 (0.33 - 0.42) | -1.48 | No |

Pooled, class-level estimate; N, number of reports; ROR, reporting odds ratio; CI, confidence interval; IC_025_, lower bound of the 95% credibility interval for the Information Component; JAKi, Janus kinase inhibitors; TNFi, tumor necrosis factor inhibitors; PDE4i, phosphodiesterase 4 inhibitors.

| Supplementary Table S9. Calendar period-stratified analysis of skin cancer signals for interleukin inhibitors with sufficient reports. | | | | | | |
| --- | --- | --- | --- | --- | --- | --- |
| Drug | Period | Years | N | ROR (95% CI) | IC_025_ | Signal |
| Ustekinumab | Early | 2009-2013 | 107 | 12.58 (10.38 - 15.24) | 3.19 | Yes |
|  | Late | 2014-2025 | 1075 | 5.04 (4.75 - 5.34) | 2.21 | Yes |
| Secukinumab | Early | 2015-2019 | 211 | 1.19 (1.04 - 1.36) | 0.05 | Yes |
|  | Late | 2020-2025 | 428 | 1.06 (0.97 - 1.16) | -0.05 | No |
| Risankizumab | Early | 2019-2023 | 854 | 6.44 (6.01 - 6.9) | 2.53 | Yes |
|  | Late | 2024-2025 | 671 | 8.68 (8.02 - 9.4) | 2.87 | Yes |

Early period, first 5 years after approval; Late period, ≥6 years after approval. ROR, reporting odds ratio; CI, confidence interval; IC025, lower bound of the 95% credibility interval for the Information Component

Supplementary Table S10. Weibull time-to-onset analysis results for interleukin inhibitors

| Drug | n | α (95% CI) | β (95% CI) | Pattern |
| --- | --- | --- | --- | --- |
| Bimekizumab | 3 |  |  | Too few cases |
| Brodalumab | 7 |  |  | Too few cases |
| Guselkumab | 15 |  |  | Too few cases |
| Ixekizumab | 19 |  |  | Too few cases |
| Secukinumab | 116 | 352.73 (295.39–412.50) | 1.10 (0.98–1.30) | Random failure |
| Tildrakizumab | 18 |  |  | Too few cases |
| Ustekinumab | 272 | 429.76 (386.05–476.50) | 1.22 (1.12–1.35) | Wear-out failure |
| Risankizumab | 392 | 344.36 (316.45–373.15) | 1.18 (1.10–1.28) | Wear-out failure |
| Random failure: β not significantly different from 1, indicating approximately constant reporting over time. Wear-out failure: β > 1, reflecting a statistical trend of an increasing reporting pattern over time within this dataset rather than definitive biological cumulative toxicity.. Too few cases: fewer than 20 cases, an insufficient sample size for stable parameter estimation and algorithm convergence based on established methodologies. | | | | |

Supplementary Table S11. Co-medication analysis of interleukin inhibitors with concomitant drugs associated with skin cancer

| Drug | N | ROR (95% CI) | IC (95% CrI) | Ω_025_ with risankizumab | Ω_025_ with secukinumab | Ω_025_ with ustekinumab |
| --- | --- | --- | --- | --- | --- | --- |
| Azathioprine | 268 | 10.59 (9.38 to 11.95) | 3.38 (3.15 to 3.51) | NA | -5.38 | -2.97 |
| Cyclosporine | 687 | 3.29 (3.05 to 3.54) | 1.70 (1.59 to 1.81) | -0.34 | -1.16 | -0.78 |
| Hydrochlorothiazide | 198 | 6.03 (5.22 to 6.91) | 2.57 (2.33 to 2.74) | -0.94 | -2.70 | -1.47 |
| Infliximab | 4313 | 5.03 (4.88 to 5.19) | 2.25 (2.20 to 2.29) | -4.41 | -3.40 | -2.08 |
| Tacrolimus | 1815 | 4.77 (4.55 to 5.00) | 2.23 (2.16 to 2.29) | NA | -1.48 | -1.09 |
| Methotrexate | 1745 | 2.57 (2.45 to 2.69) | 1.34 (1.27 to 1.40) | -2.54 | -1.80 | -1.18 |
| Apremilast | 335 | 0.98 (0.88 to 1.09) | -0.03 (-0.18 to 0.13) | -1.10 | -0.44 | -0.87 |
| ROR, reporting odds ratio; IC, information component; Ω_025_, lower bound of the 95% credibility interval of the Ω shrinkage measure; NA, not available. | | | | | | |

Supplementary Table S12. The READUS-PV checklist

| **Section and topic** | **Item #** | **Checklist item** | **Location where item is reported** |
| --- | --- | --- | --- |
| **Title** |  |  |  |
|  | *1a* | *If disproportionality analyses are a prominent component of the published study, the study should be identified as a “disproportionality analysis”. The type of data and name of the database(s) should be specified.* | 1 |
|  | *1b* | *Report the name of adverse event(s) and/or drug(s) under study, when applicable.* | 1 |
| **Introduction** |  |  |  |
| Background | *2a* | *Describe the drug(s) and its utilization, the nature of the adverse event(s) under study and its frequency, and the existing knowledge on the drug-event combination.* | 2,3 |
|  | *2b* | *Specify the rationale for performing the analysis, e.g., as part of routine pharmacovigilance, to investigate an overall safety profile, or to assess a pre-specified hypothesis.* | 3 |
|  | *2c* | *Explain why ICSR databases and disproportionality analysis are suitable to fill the knowledge gap.* | 3 |
| Objectives | *3* | *State specific objectives, identifying the adverse event(s), the drug(s), and the reference group, including any pre-specified hypothesis, if applicable.* | 3,4 |
| **Methods** |  |  |  |
| Study design | *4a* | *Identify the study (i.e., “disproportionality analysis”) and the type of data used (e.g., “individual case safety reports”).* | 4 |
|  | *4b* | *Provide an outline of the entire study design, including primary and sensitivity analyses performed, and other designs such as case-by-case analysis or literature review.* | 4,5 |
| Data description, access, and pre-processing | *5a* | *Specify the name of the database(s), the database(s) custodian, and the coverage. Specify the type/number of drugs included within the database and the thesaurus, taxonomies, or ontologies used for coding drugs and events.* | 4 |
|  | *5b* | *Specify the extraction dates and describe and justify all choices used for data pre-processing, including any data transformation or exclusion, if appropriate.* | 4 |
| Variables definition | *6a* | *Describe the study population, including any restriction.* | 4 |
|  | *6b* | *Describe the nature and the meaning of key variables assessed in the work.* | 4 |
|  | *6c* | *Specify and justify any grouping of drugs or events. For drugs, specify and justify whether active ingredients/trade names/salts were considered and/or the selected role.* | 4 |
|  | *6d* | *Describe any additional data source used, the type of data, and how they interact with ICSRs.* | NA (only FAERS data used) |
| Statistical methods | *7a* | *Present any descriptive analysis performed, specifying variables investigated, statistical tests, and significance thresholds.* | 5 |
|  | *7b* | *Describe the measure(s) selected for the disproportionality analysis including any threshold used to identify signals of disproportionate reporting. Explain the reason for this choice if applicable.* | 5 |
|  | *7c* | *Clearly describe any sensitivity analysis and any tool to control confounding, including any restriction, subgroup, stratification, adjustment, or interaction.* | 5 |
|  | *7d* | *Specify the variables and methods used for the case-by-case analysis, including any algorithm or criteria used to assess causality, if performed.* | NA (not performed) |
|  | *7e* | *Specify any statistical methods used for other data sources.* | NA (not applicable) |
| **Results** |  |  |  |
| Participants | *8a* | *Specify the number of individual case safety reports included at each stage, including reasons for exclusion.* | 6 |
|  | *8b* | *Provide key demographic and clinical characteristics of cases, if possible comparing cases with any appropriate reference group.* | 6 |
| Disproportionality analysis | *9* | *Present all results including confidence intervals. Present also results of sensitivity analyses, if performed.* | 6 |
| Case-by-case analysis | *10* | *Present the case-by-case analysis of key variables. Present the causality assessment, if applicable.* | NA (not performed) |
| **Discussion** |  |  |  |
| Key results | *11* | *Discuss key results with reference to study objectives and contextualize them within the current literature and other consulted sources. Clearly discriminate between expected reactions and emerging safety signals.* | 8 |
| External validity | *12a* | *Discuss the external validity of the results to the general population.* | 9,10 |
|  | *12b* | *Discuss the potential relevance of results in clinical practice* | 11 |
|  | *12c* | *Propose further study designs if applicable* | 11 |
| Limitations | *13* | *Present general limitations, making clear that disproportionality analysis alone cannot prove causation or measure incidence, and specific limitations, including confounding and reporting bias and efforts to mitigate them.* | 11 |
| **Declarations** |  |  |  |
|  | *14a* | *Provide the source of funding/sponsorship and the role of the funders/sponsors for the present study and for any original study on which the present article is based.* | 12 |
|  | *14b* | *Clearly identify potential commercial and intellectual conflicts of interest (e.g., link to any drug/event investigated, whether financial, legal action, or software used).* | 12 |
|  | *14c* | *Declare any institutional approval needed or granted in the investigation.* | 12 |
|  | *14d* | *Include a statement on data availability, code availability (including the version of the statistical software used), and protocol registration.* | 12 |

"NA" in items 6d, 7d, 7e, and 10 indicates not applicable or not performed in the present study.
